# Evaluation of the implementation of WHO infection prevention and control core components in Turkish health care facilities: results from a WHO infection prevention and control assessment framework (IPCAF)—based survey

**DOI:** 10.1186/s13756-023-01208-0

**Published:** 2023-02-13

**Authors:** Emel Azak, Ahmet Sertcelik, Gulden Ersoz, Guven Celebi, Fatma Eser, Ayse Batirel, Yasemin Cag, Zeynep Ture, Derya Ozturk Engin, Meltem Arzu Yetkin, Sedat Kaygusuz, Aslıhan Candevir, Ermira Tartari, Jordi Rello, Emine Alp, THIRG, Turkish Hospital Infection Research Group, Ali Seydi Alpay, Arzu Altuncekic Yildirim, Asli Vatan, Aysun Yahsi, Ayse Kaya Kalem, Ayse Sagmak Tartar, Aysegul Tuna, Banu Karaca, Belgin Coskun, Burcu Gonulal, Canan Demir, Davut Ipek, Dilsat Tepe, Duru Mıstanoglu Ozatag, Edanur Sezer, Emine Sehmen, Emine Unal Evren, Emsal Aydın, Ertugrul Guclu, Esma Eryilmaz Eren, Esmeray Mutlu Yilmaz, Fatma Yilmaz Karadag, Ferhan Kerget, Filiz Surucu Bayar, Gamze Kalin Unuvar, Gulden Eser Karlidag, Gulfem Akengin Ocal, Gulnur Kul, Gunes Senol, Gurdal Yilmaz, Haluk Erdogan, Handan Alay, Hande Arslan, Hasip Kahraman, Hatun Ozturk Cerik, Hulya Caskurlu, Ilknur Erdem, Ilknur Esen Yildiz, Kivanc Serefhanoglu, Kubra Demir Onder, Lutfiye Nilsun Altunal, Mehmet Celik, Mehmet Resat Ceylan, Merve Sefa Sayar, Metehan Ozen, Muharrem Guler, Mustafa Uguz, Mustafa Yildirim, Mucahide Esra Kocoglu, Muge Ayhan, Muge Toygar Deniz, Nagehan Didem Sari, Nazan Tuna, Nevin Ince, Ozlem Bayrak, Oznur Ak, Ramazan Gozukuçuk, Recep Balik, Salih Atakan Nemli, Selda Aslan, Selma Ilkay Sahin, Semiha Solak Grassie, Serpil Unlu, Sevil Alkan, Sibel Altunisik Toplu, Suna Secil Ozturk Deniz, Suheyla Komur, Suleyman Koc, Saban Incecik, Tuba Yanik Yalcin, Tuna Demirdal, Turkan Tuzun, Verda Dinar Tuna, Yasemin Cakir, Yasemin Ersozlu, Yesim Aybar Bilir, Yesim Uygun Kizmaz, Yildiz Olcar, Zerrin Yulugkural

**Affiliations:** 1 Department of Infectious Diseases and Clinical Microbiology, Faculty of Medicine, Kocaeli University, Kocaeli, Türkiye; 2 Division of Epidemiology, Department of Public Health, Faculty of Medicine, Hacettepe University, Ankara, Türkiye; 3 Department of Infectious Diseases and Clinical Microbiology, Faculty of Medicine, Mersin University, Mersin, Türkiye; 4 Department of Infectious Diseases and Clinical Microbiology, Zonguldak Bulent Ecevit University Faculty of Medicine, Zonguldak, Türkiye; 5 Department of Infectious Diseases and Clinical Microbiology, Ankara Yildirim Beyazit University, Ankara, Türkiye; 6 Department of Infectious Diseases and Clinical Microbiology, University of Health Sciences, Kartal Dr. Lutfi Kirdar City Hospital, Istanbul, Türkiye; 7 Department of Infectious Diseases and Clinical Microbiology, Istanbul Medeniyet University Faculty of Medicine, Istanbul, Türkiye; 8 Department of Infectious Diseases and Clinical Microbiology, Erciyes University Faculty of Medicine, Kayseri, Türkiye; 9 Department of Infectious Diseases and Clinical Microbiology, Sancaktepe Sehit Prof. Dr. Ilhan Varank Training and Research Hospital, Istanbul, Türkiye; 10 Department of Infectious Diseases and Clinical Microbiology, Giresun University Faculty of Medicine, Giresun, Türkiye; 11 Department of Infectious Diseases and Clinical Microbiology, Kirikkale University Faculty of Medicine, Kirikkale, Türkiye; 12 Department of Infectious Diseases and Clinical Microbiology, Cukurova University Faculty of Medicine, Adana, Türkiye; 13 Faculty of Health Sciences, University of Malta, Msida, Malta; 14 Catedràtic de Medicina, Universitat Internacional de Catalunya, Barcelona, Spain

**Keywords:** Infection prevention and control, IPC core components, Health care-associated infections, Antimicrobial resistance, Workload

## Abstract

**Background:**

The core components (CCs) of infection prevention and control (IPC) from World Health Organization (WHO) are crucial for the safety and quality of health care. Our objective was to examine the level of implementation of WHO infection prevention and control core components (IPC CC) in a developing country. We also aimed to evaluate health care-associated infections (HAIs) and antimicrobial resistance (AMR) in intensive care units (ICUs) in association with implemented IPC CCs.

**Methods:**

Members of the Turkish Infectious Diseases and Clinical Microbiology Specialization Association (EKMUD) were invited to the study via e-mail. Volunteer members of any healt care facilities (HCFs) participated in the study. The investigating doctor of each HCF filled out a questionnaire to collect data on IPC implementations, including the Infection Prevention and Control Assessment Framework (IPCAF) and HAIs/AMR in ICUs in 2021.

**Results:**

A total of 68 HCFs from seven regions in Türkiye and the Turkish Republic of Northern Cyprus participated while 85% of these were tertiary care hospitals. Fifty (73.5%) HCFs had advanced IPC level, whereas 16 (23.5%) of the 68 hospitals had intermediate IPC levels. The hospitals’ median (IQR) IPCAF score was 668.8 (125.0) points. Workload, staffing and occupancy (CC7; median 70 points) and multimodal strategies (CC5; median 75 points) had the lowest scores. The limited number of nurses were the most important problems. Hospitals with a bed capacity of > 1000 beds had higher rates of HAIs. Certified IPC specialists, frequent feedback, and enough nurses reduced HAIs. The most common HAIs were central line-associated blood stream infections. Most HAIs were caused by gram negative bacteria, which have a high AMR.

**Conclusions:**

Most HCFs had an advanced level of IPC implementation, for which staffing was an important driver. To further improve care quality and ensure everyone has access to safe care, it is a key element to have enough staff, the availability of certified IPC specialists, and frequent feedback. Although there is a significant decrease in HAI rates compared to previous years, HAI rates are still high and AMR is an important problem. Increasing nurses and reducing workload can prevent HAIs and AMR. Nationwide “Antibiotic Stewardship Programme” should be initiated.

**Supplementary Information:**

The online version contains supplementary material available at 10.1186/s13756-023-01208-0.

## Background

Health care-associated infections (HAIs) are one of the most common adverse events in health care [1]. Despite the developments in infection control measures today, HAIs continue to maintain their importance with their incidence, mortality, cost, and contribution to antimicrobial resistance [2–6]. The HAI burden is reported to be higher in low- and middle-income countries (LMICs) than in developed countries [2, 7]. It is estimated that 63.5% of infections caused by antibiotic-resistant bacteria are health care-related [6]. In recent years, although carbapenem resistance in *Acinetobacter*, *Klebsiella*, and *Pseudomonas* species has been an important problem, methicillin resistance in *Staphylococcus aureus* and vancomycin resistance in *Enterococcus faecium* continue to maintain their importance [8–11]. The major high-risk areas for HAIs in healthcare facilities are intensive care units (ICU). Both HAI and antimicrobial resistance (AMR) rates are reported to be higher in patients hospitalized in the ICU [12]. The mortality ratio in patients infected with resistant microorganisms is at least two to three times higher than in patients infected with susceptible microorganisms [2]. Therefore, prevention of HAIs is a global priority in order to reduce AMR, which has been going on for years, the so-called “silent pandemic”, and other adverse effects on the health system. Infection prevention and control (IPC) measures and antimicrobial stewardship (AMS) programmes that will prevent the development of HAI and AMR gain more importance for countries with limited resources due to the fact that they constitute a significant burden on the country's economy [13].

In the SENIC study, performed in the early 1970s to assess the efficiency of the control of nosocomial infections, the establishment of surveillance and/or infection control measures was shown to prevent HAIs, and practices aimed at preventing HAIs gained momentum [14].

In 2016, the World Health Organization (WHO) made recommendations for IPC programmes, summarized in eight core components (CCs), and subsequently specified the minimum requirements for IPC practices in 2018 [15, 16]. In addition, the WHO released the Infection Prevention and Control Assessment Framework (IPCAF) questionnaire [17].

IPC minimum requirements consist of eight CC, including the IPC programme (CC1), IPC guidelines (CC2), IPC education and training (CC3), HAI surveillance (CC4), multimodal strategies for implementing IPC activities (CC5), monitoring and audits of IPC practices and feedback (CC6), workload, staffing, and occupancy (CC7), and the built environment, materials, and equipment for IPC at the facility level (CC8) [15]. In the IPCAF survey, establishments are given a score based on these eight CCs, and the results are then classified into one of the four IPC categories. Insufficient is indicated by a score of 0–200 points; basic is 201–400 points; intermediate is 401–600 points; and advanced IPC level is 601–800 points [17]. In addition, this survey provides a possibility for evaluation, analysis, and improvement of IPC activities carried out in health care facilities (HCFs).

The COVID-19 pandemic we are experiencing has shown how necessary IPC programmes are for the safety of corporate patients and health care professionals, as well as global health security [18]. With the implementation of IPC programmes, safe and quality health care can be provided for those who use health services and those who offer this service [19].

Until the 2000s, IPC activities in Türkiye were carried out through individual efforts spearheaded by academic members. The most crucial step to strengthening IPC activities in Türkiye has been achieved with the Inpatient Treatment Institutions Infection Control Regulation published in 2005 [20]. With this regulation, national surveillance and IPC programmes supported by the Ministry of Health have been initiated. Infection control committees (ICC) with at least one certified full-time infection control nurse and infection control doctor in all institutions have been established, national surveillance ground rules have been developed, and then multimodal hand hygiene campaigns and national education and training programmes have been carried out. In 2006, HAI and hand hygiene compliance data started to be provided to a national data server. In 2008, surveillance data began to be reported to the Ministry of Health via a web-based surveillance system (the National Health Service Associated Infections Surveillance Network, or USHIESA) using internationally accepted definitions and standardized forms [20–22]. National HAI surveillance has focused primarily on the surveillance and prevention of device-associated HAI (DA-HAI) in ICUs [23]. With the change in the Infection Control Regulation of Inpatient Treatment Institutions in 2011, assigning an infection control nurse for every 150 full beds became mandatory, taking into account the annual average bed occupancy rate [20].

Trainings for infection control nurses were initiated by the Ministry of Health in 2007 with a standard training programme. Trainings were provided at Ministry-approved training centers until 2017. Since 2017, the theoretical part of the education has been carried out using distance education techniques, followed by practical training lasting 15 working days at the training centers. For theoretical education, 40 lessons in 7 modules are taught by 22 trainers. For 2018, the total number of training centers in 17 provinces is 37. The validity period of the infection control nursing certificate is seven years. The certificates of nurses whose certificates have expired are renewed through the recertification exam, which has been offered via distance education since 2017. Certified nurses can benefit from distance theoretical education. Until 2013, a training programme given by the Ministry for physicians working in infection control committees, followed by an infection control doctor certification exam, was implemented. Infection control doctor certification has been abolished since 2013. All physicians can participate in infection control trainings, which started in 2017 and are conducted using distance education techniques, and receive participation certificates when they complete the training [21, 22].

Nearly 15 years have passed since the start of the national IPC program in Türkiye. However, the degree of implementation of IPC CCs in Turkish HCFs and IPC practices’ effect on HAI rates have not been investigated yet.

The main aim of this study is to evaluate the IPC practices in Turkish HCFs and determine the level of IPC in Turkish HCFs using the IPCAF survey tool. Our second aim is to determine how often HAIs and AMR occur in ICUs in 2021 and whether the lack of IPC components is usually associated with HAIs.

## Methods

### Study design and participants

Members of the Türkiye Infectious Diseases and Clinical Microbiology Specialization Association (EKMUD) dealing with HAIs were invited to the study via e-mail. EKMUD is the national, professional, and scientific association of infectious diseases and clinical microbiology specialists. According to the statistics of the ministry of health in Türkiye for 2020, there are a total of 1534 hospitals in Türkiye, including 900 hospitals under the ministry of health, 68 university hospitals, and 566 private hospitals [24]. In Turkish Republic of Northern Cyprus (TRNC), there are two university hospitals and four private hospitals. Members of the EKMUD are present in most hospitals in Türkiye and the TRNC. The participation of the centers was based on voluntarism, and there were no criteria other than involvement with HAIs. A questionnaire form was created for the study. The first part of the questionnaire was a self-assessment, which included the descriptive characteristics of HCFs (hospital type, units available, number of beds, number of doctors and nurses in ICUs and HCFs, number of infection control doctors and nurses, etc.) and information about IPC CCs, including the IPCAF questionnaire for 2021. The questionnaire was in Turkish, and the IPCAF questionnaire was translated into Turkish. The Turkish version of the IPCAF questionnaire is shown in Additional File 1. The second part of the questionnaire included data on HAIs, causative pathogens, and resistance to selected antibiotics in ICU in 2021. Centers for Disease Control and Prevention (CDC) /National Healthcare Safety Network (NHSN) criteria were used to define HAIs [25]. For HAI developing in the ICU, infections developing on or after the 3rd day of admission to the ICU or the transfer rule in the CDC/NHSN criteria were considered [15]. Pathogens that caused HAIs in ICUs were tested to see how resistant they were to certain antibiotics. Before the study data were collected, online meetings were held with all participants to go over the study's design and the introduction of the questionnaire forms in order to get reliable results.

In order to ensure data confidentiality, a special code was created for each institution in the study, and each HCF provided data entry with this code. Google Forms was used to collect all data.

Each center separately obtained administrative permission for the study, which was ethically approved by the Kocaeli University Faculty of Medicine Non-Invasive Clinical Research Ethics Committee (GOKAEK-2022/01.19, project number: 2022/15).

### Measurements

According to the answers given to each question in the IPCAF questionnaire, the scoring specified in the guide was applied, and then a descriptive analysis was performed. It was possible to get a maximum of 100 points for each of the eight CCs of the IPCAF. After adding up the eight CC scores, the maximum IPCAF score was 800. According to the final total score, hospitals were categorized into four different IPC levels: 0–200 points ‘‘insufficient’’, 201–400 points ‘‘basic’’, 401–600 points ‘‘intermediate’’, and 601–800 points ‘‘advanced’’.

### Statistical analysis

SPSS software (version 21; IBM, Chicago, IL, USA) was used for statistical analysis. Descriptive statistics were presented as numbers and percentages, mean ± standard deviation (SD) if quantitative data fit the normal distribution, and median, interquartile range (IQR) if they did not. Compliance with the quantified normal distribution was evaluated with histogram, Detrended Q-Q plot, and Shapiro–Wilk test. Comparisons (Chi-square, Fischer's exact test, Student's t-test, Mann Whitney U test) and multivariate analysis were performed to identify factors associated with rates of HAIs. The significance level was accepted as *p* < 0.05 (bi-directional). No imputations were made to replace the missing values.

## Results

### Characteristics of participating hospitals

**Fig. 1:**
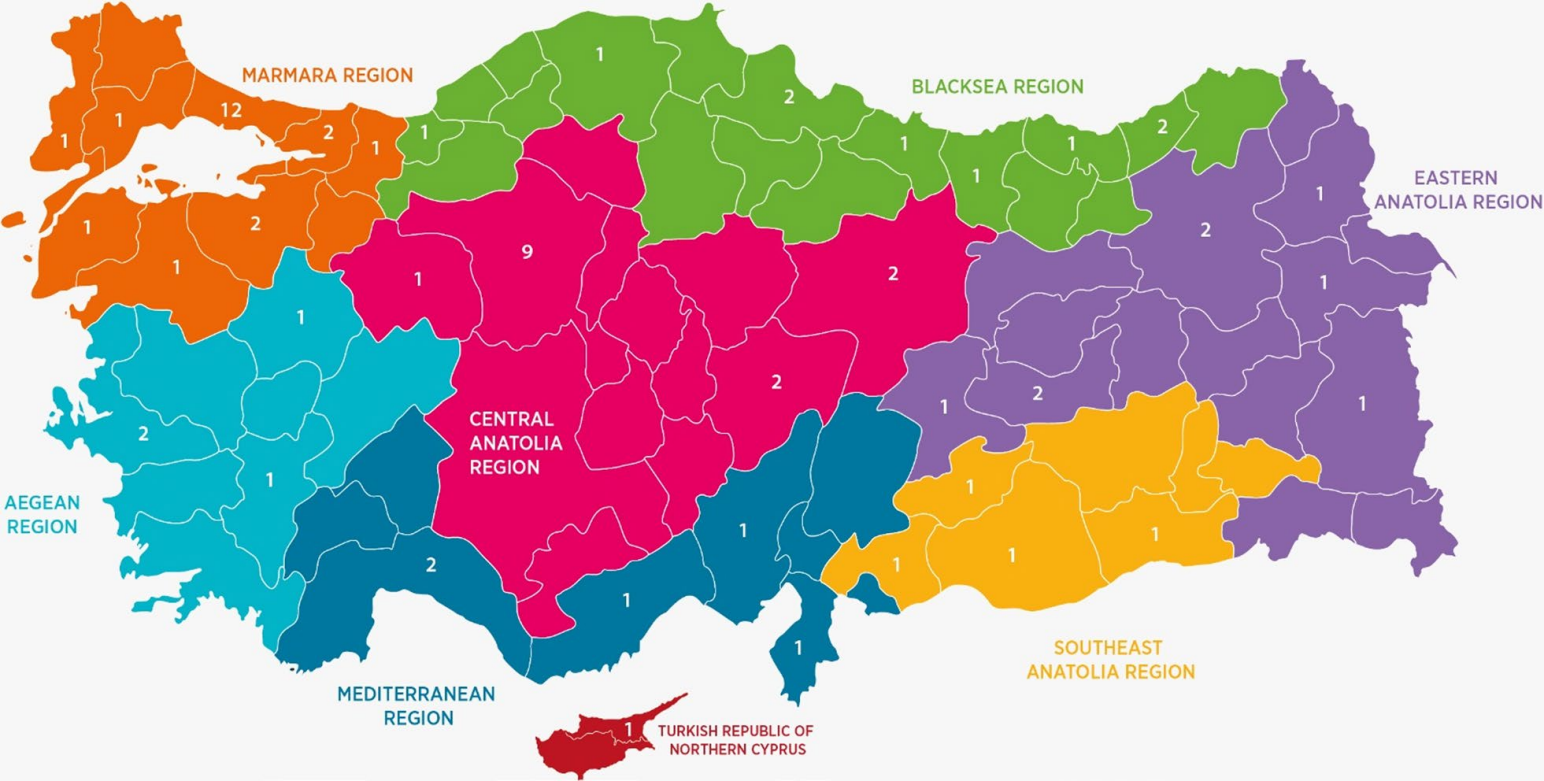
Distribution of the hospitals included in the study to the seven regions of Türkiye and the Turkish Republic of Northern Cyprus

In total, there are 1536 HCFs in Türkiye and 6 HCFs in the TRNC. At the first stage, 76 of these HCFs stated that they were willing to participate in the study [24]. Eight HCFs subsequently withdrew from the study, stating that they did not have sufficient time to collect and present data. Therefore, the study was carried out at 68 HCFs. 85% of these are hospitals classified as tertiary care. The distribution of these hospitals was training and research hospitals (TRH, 39.7%), university hospitals (UH, 32.4%), and city hospitals (CH, 13.2%). In addition, seven of the other HCFs participating in the study were state hospitals (SH, 10.3%), and only three were private hospitals (PH, 4.4%). The distribution of HCFs included in the study from Türkiye and the TRNC is shown in Fig. 1.

### The distribution of IPCAF scores

**Fig. 2:**
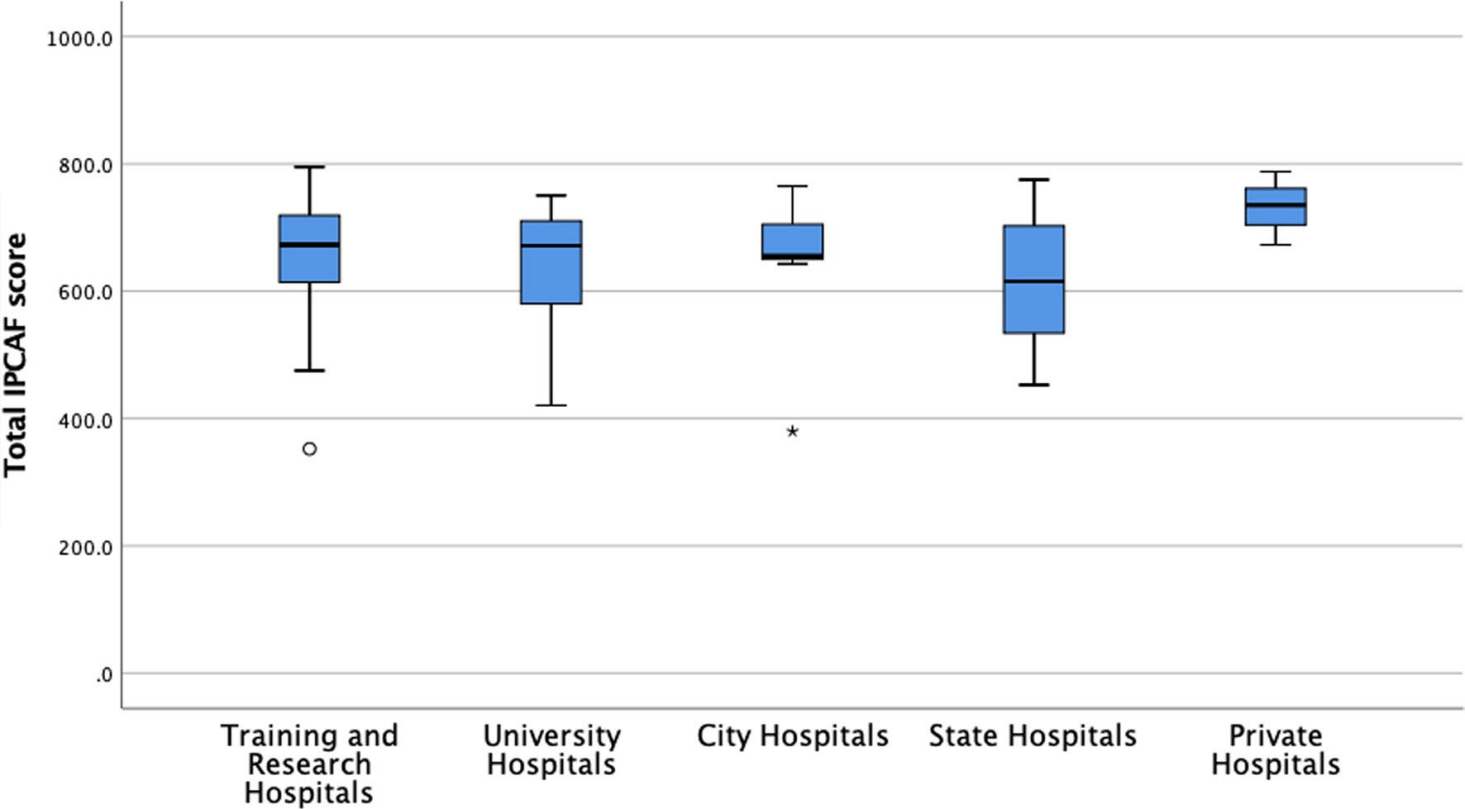
The evaluation of participating hospitals according to IPCAF score results

When the IPCAF results from 68 HCFs were analyzed, it was found that 0 HCF had inadequate IPC levels, 2 (2.9%) had basic IPC levels, 16 (23.5%) had intermediate levels, and 50 (73.5%) had advanced levels. The median (IQR) IPCAF score of the HCFs was 668.8 (125.0) points. Advanced IPC levels were present in 77.8% of TRHs, 59.1% of UHs, 88.9% of CHs, 71.4% of SHs, and 100% of PHs (Fig. 2).

### IPC core components

IPC guidelines (CC2) had the highest scores, while multi-modal strategies (CC5) and workload, staffing, and bed occupancy at the facility level (CC7) had the lowest scores (Table 1).

**Table 1:** Distribution of results of the total IPCAF scores among participating hospitals, Median (IQR)

Component	Score					
	TRH (n = 27)	UH (n = 22)	CH (n = 9)	SH (n = 7)	PH (n = 3)	Total (n = 68)
CC1	82.5 (17.5)	90.0 (15.6)	90.0 (23.8)	80.0 (45.0)	100.0 (-)	90.0 (17.5)
CC2	97.5 (10.0)	98.8 (15.0)	100.0 (12.5)	100.0 (35.0)	97.5 (-)	98.8 (13.8)
CC3	85.0 (25.0)	85.0 (30.0)	75.0 (20.0)	70.0 (50.0)	90.0 (-)	80.8 (20.0)
CC4	92.5 (17.5)	91.3 (8.1)	90.0 (26.3)	82.5 (27.5)	100.0 (-)	92.5 (12.5)
CC5	80.0 (50.9)	75.0 (48.0)	75.0 (28.0)	65.0 (80.0)	85.0 (-)	75.0 (39.0)
CC6	90.0 (10.0)	90.0 (11.3)	85.0 (6.3)	87.5 (20.0)	97.5 (-)	90.0 (11.9)
CC7	65.0 (20.0)	62.5 (39.0)	75.0 (20.0)	75.0 (15.0)	90.0 (-)	70.0 (34.0)
CC8	95.0 (17.5)	81.2 (28.8)	95.0 (13.8)	87.5 (12.5)	92.5 (-)	92.5 (20.0)
Total	672.5 (115.0)	671.3 (140.0)	655.0 (71.3)	615.0 (300.0)	735.0 (-)	668.8 (125.0)

*TRH* training and research hospital, *UH* university hospital, *CH* city hospital, *SH* state hospital, *PH* private hospital, *IPCAF* Infection Prevention and Control Assessment Framework, *CC* core component, *CC1* IPC programme, *CC2* IPC guidelines, *CC3* IPC education and training, *CC4* HAI surveillance, *CC5* multimodal strategies for implementing IPC activities, *CC6* monitoring/audits of IPC practices and feedback, *CC7* workload, staffing and occupancy, *CC8* built environment, materials and equipment for IPC at the facility level, *IPC* Infection Prevention Control, IPCAF Infection Prevention Control Assessment Framework

#### CC1: IPC programme

When the IPCAF score for CC1 of all HCFs was evaluated, the median (IQR) CC1 score was 90.0 (17.5) points. CC1 scores were high in PH, UH, and CH but low in TRH and SH. However, more varied results were seen when focusing on specific questions in CC1. It was determined that only 15 (22%) of the hospitals had a budget allocated for the IPC programme, although senior institution leaders reported that 74% of the hospitals supported the IPC targets and indicators within their hospital. Only 47.1% of all HCFs met the WHO recommendation that there be one IPC specialist for every 250 beds. In addition, in 74% of the hospitals, the number of infection control nurses was below the number recommended by the national regulation (1 nurse/150 beds).

#### CC2: IPC guidelines

The IPCAF score for CC2 of all HCFs was the highest among all CCs, with a median (IQR) of 98.8 (13.8) points. The IPCAF score for CC2 of all HCFs was the highest among all CCs, with a median (IQR) of 98.8 (13.8) points. All institutions said they had written IPC guidelines. These guidelines are essentially national guidelines. Some of the guidelines have been regulated locally on the basis of national and international guidelines. But when specific guidelines were looked at, the results were mixed. Despite the COVID-19 pandemic, 5 (7.4%) of the HCFs still do not have guidelines for handling outbreaks and being ready for them. Antibiotic stewardship (85.3%) and injection safety (86.8%) were written guidelines with relatively lower rates than other guidelines.

#### CC3: IPC education and training

The median (IQR) IPCAF score for CC3 of all HCFs was 80.8 (20.0) points. All of the HCFs reported that they have a training programme on hand hygiene, isolation precautions, environmental cleaning and disinfection, and the use of personal protective equipment, and that training is provided at least once a year. Other training topics are catheter-associated urinary tract infection (CA-UTI) prevention (95.6%), central line-associated blood stream infections (CLABSI) prevention (97.1%), ventilator-associated pneumonia (VAP) prevention (94.1%), waste management (95.6%), instrument cleaning, disinfection, and sterilization (97.1%), surgical site infection (SSI) prevention (92.6%), and personnel health (79.4%). While 48.5% of these trainings were only with written information, verbal instruction, or e-learning, 51.5% were additional interactive training sessions (e.g., simulation and/or bedside training).

Besides the IPC professionals, 45 (66.2%) of the HCFs had staff with enough skills to help with education. Only 22.1% of the HCFs made sure that IPC training was part of clinical practice and training for all specialties. Also, it was found that patients and family members get less IPC training than health care workers.

#### CC4: HAI surveillance

The median (IQR) IPCAF score for CC4 of all HCFs was 92.5 (12.5) points. Surveillance was applied both on a clinical (clinical identification of nosocomial infections based on clinical symptoms and signs in the absence of microbiological testing) and laboratory basis in 98.5% of hospitals. Seven (10.3%) of the hospitals said they didn't have enough information technology (IT) help to handle surveillance.

#### CC5: Multimodal strategies for implementing IPC activities

The median (IQR) IPCAF score for CC5 across all HCFs was 75 (12.5) points, among the lowest scores. 48 HCFs (70.6%) stated that they used multimodal strategies.

#### CC6: Monitoring, evaluation and feedback

The median (IQR) IPCAF score for CC6 of all HCFs was 90 (11.9) points. In 98.5% of HCFs, monitoring data was reported regularly (at least once a year).

#### CC7: Workload, staffing, and bed occupancy at the facility level

The IPCAF score for CC7 of all centers was the lowest, with a median (IQR) of 70 [34] points. Most HCFs stated that they do not use standard assessment tools (WHO or national) for workload and staffing levels and that there is no system in place to act on the results of staffing needs assessments when staffing levels are deemed to be insufficient. In our additional evaluations, the limited number of nurses and their workload were identified as the most important problems. The ratio of nurses to patients in the tertiary ICUs was greater than 0.5 for 62% of the day shift and 47% of the night shift.

#### CC8: Built environment, materials, and equipment for IPC at the facility level

The median (IQR) IPCAF score for CC8 of all HCFswas 92.5 [20] points. Most HCFs reported that the built environment, materials, and equipment for IPC at the facility level were available and sufficient.

A full description of all the IPCAF questions and the answers we received from the participating hospitals can be found in Additional File 2 of this article.

### HAIs and AMR in ICUs

The features of ICUs and HAIs, the microorganisms found in HAIs in ICUs, and the rates of AMR to selected antibiotics in the HAI-causing pathogens are detailed in Tables 2, 3, and 4 for 2021 in these ICUs. Also, the features of ICUs and HAIs in ICUs can be found in detail according to HCF types in Additional File 3.

**Table 2:** The features of ICUs and HAIs in ICU, 2021

Features of ICUs	Adult ICU	Neonatal ICU	Pediatric ICU
Number of centers performing intensive care unit surveillance	64	47	38
Number of hospital beds	44,845	37,916	33,062
Number of hospitalized patients	2,193,219	1,886,744	1,635,103
Number of ICU beds	5579	1597	580
Number of nurses per day bed, Median (IQR)	0.50 (0.05)	0.33 (0.25)	0.50 (0.15)
Number of nurses per bed per night, (Median (IQR)	0.50 (0.17)	0.33 (0.25)	0.50 (0.17)
Number of patients hospitalized in the ICU	247,039	51,003	16,274
Patient days in ICU	1,241,011	402,211	142,799
Number of infected patients in ICU	18,276	979	649
Number of nosocomial infections in ICU	11,969	1237	806
Number, Non-device-associated nosocomial infection in ICU	3161	649	259
Number, Device-associated nosocomial infection in ICU	8808	588	547
**Device utilization ratio**			
Central line utilization ratio	0.46	0.2	0.5
Urinary catheter utilization ratio	0.88	0.007	0.3
Ventilator utilization ratio	0.36	0.17	0.5
**Incidence density of device-associated HAI (per 1000 device days)**			
CLABSI	7.1	6.9	4.9
CA-UTI	0.13	2.8	0.9
VAP	4.8	1.9	2.1
VAE	2,8		
**Non-device-associated HAI in ICU (per 1000 patients days)**			
BSI	0.12	0.9	0.8
UTI	0.3	0.2	0.2
Pneumonia	0.8	0.06	0.2
LRI	0.04	0.003	0.04
Skin and soft tissue infection	0.15	0.05	0.2
Bone and joint infection	0.009	0	0
Cardiovascular system infection	0.005	0.003	0.01
Eye, ear, nose, throat and mouth infections	0.015	0.1	0.06
CNI	0.05	0.1	0.1
GI	0.03	0.2	0.04
RI	0.005	0	0
Systemic infections	0.0008	0.01	0.03

*ICU* Intensive care unit, *HAI* Health care-associated infection, *TRH* training and research hospital, *UH* university hospital, *CH* city hospital, *SH* state hospital, *PH* private hospital, *CLABSI* Central line- associated blood stream infections, *CA-UTI* Catheter-associated urinary tract infection, *VAE* Ventilator-associated event, *VAP* Ventilator-associated pneumonia, *BSI* blood stream infections*, UTI* urinary tract infection*, LRI* Lower respiratory tract infection, other than pneumonia, *CNI* Central nervous system infection, *GI* Gastrointestinal system infection, *RI* Reproductive tract infection, *IQR* interquartile range

**Table 3:** The microorganisms found in HAIs in ICUs, %

	Adult ICU						Neonatal ICU						Pediatric ICU					
Organism/group	TRH	UH	CH	SH	PH	Total	TRH	UH	CH	SH	PH	Total	TRH	UH	CH	SH	PH	Total
**Gram-negative bacteria**	**68.6**	**77.4**	**60.7**	**72.6**	**90**	**70.6**	**62.4**	**69.5**	**70.9**	**55.2**	**60**	**66.4**	**74.5**	**74.9**	**62.5**	**83.7**	**100**	**70.8**
*Acinetobacter baumannii*	20.3	20.6	26.1	18.8	14.5	21.2	5.7	10.98	15.6	20.7	0	9.5	4.3	14.3	9.1	28.6	33.3	12.16
*Acinetobacter* spp.	3	5.6	0.1	5.9	0.1	3.2	1.1	1	0	0	0	0.8	0	2	0.6	0	0	1.2
*Pseudomonas aeruginosa*	5.7	9.4	5.1	7.2	10.7	6.9	4.4	5.1	7.8	0	20	5.8	19.2	15.2	14.7	28.6	33.3	16.2
*Pseudomonas* spp.	1.6	2.4	0.1	1.6	0	1.4	0.5	0.8	0.7	0	0	0.7	1.1	2	0.3	0	0	1.2
*Klebsiella pneumoniae*	11.1	20.9	18.8	15.5	25.7	16.3	19.1	22.6	25.5	20.7	36.4	22.4	15.96	24.4	22.3	26.5	33.3	22.9
*Klebsiella* spp.	8.8	0.8	0.3	3.1	3.8	4.3	3.3	3.5	3.6	0	0	3.1	2.1	0.7	0.3	0	0	0.7
*Escherichia coli*	4.5	6.34	4	15.3	26.4	7	4.4	5.5	7.8	6.9	1.8	5.3	1.1	2.3	5.9	0	0	3.3
*Stenotrophomonas maltophilia*	4.6	4.9	3.3	1.2	0.5	3.9	3.8	7.9	1.4	0	1.8	5.2	14.9	8.1	3.5	0	0	6.7
*Proteus* spp.	6.1	2.7	0.9	*0.8*	4.6	3.6	0.8	0.4	0	0	0	0.5	5.3	1.8	1.8	0	0	2.1
*Enterobacter* spp.	1	1.9	1.4	0.5	3.8	1.4	7.1	4.7	2.1	3.5	0	4.9	0	0.9	1.5	0	0	1
*Other Enterobacteriaceae*	1.3	1.4	0.6	1	0	1.1	12.3	6.9	4.3	3.5	0	7.9	10.6	3.2	2.6	0	0	3.6
*Other*	0.6	0.5	0	1.8	0	0.5	0	0.2	2.1	0	0	0.4	0	0	0	0	0	0
**Gram-positive bacteria**	**16.5**	**15.9**	**31.3**	**26.6**	**8.5**	**20**	**30**	**25.6**	**20.6**	**10.4**	**25.5**	**26**	**6.4**	**10.2**	**20.8**	**0**	**0**	**13.1**
*Staphylococcus aureus*	4.3	3.2	4.6	2.7	3.1	3.9	10.4	5.7	8.5	3.5	0	7.3	3.2	2.7	7.3	0	0	4.3
Coagulase-negative staphylococci	3.8	3.8	16.1	4.1	1.5	6.3	7.6	9.8	5.7	0	16.4	8.6	1.1	4.3	6.2	0	0	4.4
*Staphylococcus* spp.	0.2	0	0	11.4	0.4	1.2	0	0.4	0	0	0	0.2	0	0.2	0	0	0	0.1
*Streptococcus pneumoniae*	0.1	0.2	0.3	0.3	0.1	0.2	0	0.4	0	0	0	0.2	0	0	0	0	0	0
*Streptococcus* spp.	0.1	0.1	0.03	0.3	0.4	0.1	1.1	1.2	0	0	0	0.9	0	0	0	0	0	0
*Enterococcus faecalis*	3.5	3.2	3.6	3	1.1	3.3	6.3	3.9	2.1	0	9.1	4.6	0	2.3	3.8	0	0	2.5
*Enterococcus faecium*	4.3	4.9	6.7	2.4	1.8	4.6	4.6	3.7	4.3	3.5	0	3.9	2.1	0.2	1.8	0	0	1
*Enterococcus* spp.	0.3	0.4	0.03	2.4	0.1	0.5	0	0.6	0	3.5	0	0.4	0	0.5	1.8	0	0	0.9
*Other*	0.03	0	0	0	0	0.01	0	0	0	0	0	0	0	0	0	0	0	0
**Fungi**	**14.9**	**6.8**	**7.9**	**0.8**	**1.5**	**9.4**	**7.6**	**4.9**	**8.5**	**34.5**	**14.6**	**7.6**	**19.2**	**14.9**	**16.7**	**16.3**	**0**	**16**
*Candida albicans*	4	2.6	3.9	0.3	1	3.1	2.7	1.2	2.8	24.1	7.3	2.9	8.5	2.9	9.4	2	0	5.8
*Non-albicans Candida*	10.8	4	4	0.5	0.5	6.2	4.9	3.7	5.7	10.3	7.3	4.7	10.6	12	7.3	14.3	0	10.2
*C. auris*	0	0	0	0	0.3	0.01	0	0	0	0	0	0	0	0	0	0	0	0
*C. parapsilosis*	1.8	2.9	2.2	0.3	0.3	2	3.3	1.6	2.1	10.4	7.3	2.8	9.6	8.8	5.3	14.3	0	7.8
*C. glabrata*	0.6	0.8	0.5	0	0	0.5	1.4	0.6	2.8	0	0	1.1	0	1.1	0.9	0	0	0.9
*Candida spp. (Candida keyfr, Candida tropicalis, *etc.*)*	8.4	0.3	1.3	0.2	0	3.7	0.3	1.4	0.7	0	0	0.8	1.1	2	1.2	0	0	1.5
*Aspergillus* spp.	0.1	0.2	0	0	0	0.1	0	0	0	0	0	0	0	0	0	0	0	0

*VAP* Ventilator-associated pneumonia,* CLABSI* Central line- associated blood stream infections,* CA-UTI* Catheter-associated urinary tract infection,* HAI* Health care-associated infection,* IPC* Infection prevention and control,* TRH* Training and Research Hospitals,* UH* University Hospitals,* CH* City Hospitals,* SH* State Hospitals,* PH* Private Hospitals,* ICU* Intensive care unit, * Significant relationship between HAIs and IPC components (*p* <0.005). Bold numbers are the total number of gram positive and gram negative microorganisms

**Table 4:** The rates of antimicrobial resistance to selected antibiotics in HAIs-causing pathogens in intensive care units

Antimicrobial resistance	Microorganism	Adult ICU	Pediatric ICU	Neonatal ICU
Carbapenem resistance, %	*Acinetobacter baumannii*	92 (78–96)	89 (75–100)	94 (91–100)
	*Pseudomonas aeruginosa*	59 (30–83)	66 (0–71)	62 (0–100)
	*Klebsiella pneumoniae*	58 (35–71)	64 (46–100)	39 (10–70)
	*Escherichia coli*	10 (3–21)	26 (0–35)	6 (0–8)
Colistin resistance, %	*Acinetobacter baumannii*	7 (0–11)	11 (0–50)	3 (0–5)
	*Pseudomonas aeruginosa*	6 (1–10)	8 (0–33)	6 (0–13)
	*Klebsiella pneumoniae*	26 (8–38)	11 (0–73)	36 (3–67)
	*Escherichia coli*	4 (0–9)	0	0
ESBL positive, %	*Klebsiella pneumoniae*	57 (42–71)	75 (47–100)	57 (37–85)
	*Escherichia coli*	52 (43–71)	59 (0–100)	34 (0–50)
Oxacillin resistance, %	*Staphylococcus aureus*	43 (22–71)	44 (0–48)	37 (0–50)
	Coagulase-negative staphylococci	71 (31–79)	63 (0–100)	72 (0–88)
Teicoplanin resistance, %	*Staphylococcus aureus*	3 (0–13)	5 (0–8)	1 (0–3)
	Coagulase-negative staphylococci	9 (0–55)	15 (0–29)	6,3 (0–25)
	*Enterococcus faecium*	19 (3–39)	33 (17–100)	38 (17–100)
	*Enterococcus faecalis*	3 (0–6)	0	4 (0–11)
Vancomycin resistance, %	*Staphylococcus aureus*	2 (0–5)	0	0
	Coagulase-negative staphylococci	2 (0–55)	0	0
	*Enterococcus faecium*	14 (0–22)	33 (17–100)	43 (0–100)
	*Enterococcus faecalis*	2 (0–6)	0	2 (0–6)
Ampicillin resistance, %	*Enterococcus faecium*	83 (15–94)	100 (100)	81 (67–100)
	*Enterococcus faecalis*	14 (0–27)	13 (0–15)	10 (0–22)
Fluconazole resistance, %	*Candida albicans*	10 (0–55)	2 (0–13)	0
	Non-albicans *Candida*	16 (11–28)	10 (0–20)	16 (0–28)

*ICU* intensive care unit, *ESBL* extended spectrum beta-lactamase

### HAIs and AMR in Adult ICUs

It has been reported that a total of 247,039 inpatients developed a total of 11,969 HAIs in 64 HCFs that performed adult ICU surveillance. It was determined that the rate of urinary catheter use was very high, central line-associated blood stream infections (CLABSI) was the most common DA-HAI, and the most common non-device-associated-HAIs (NDA-HAIs) were bloodstream infections (BSI) and pneumonia. The most frequently isolated microorganisms in HAIs were *Acinetobacter baumannii* (21.2%) and *Klebsiella pneumoniae* (16.3%). It was shown that non-albicans *Candida* was more prevalent than other types of fungus. *Candida* spp. (3,7%) and *Candida parapsilosis* (2%) were the most common pathogens that were responsible for the non-albicans *Candida* infections. In only two HAIs, *Candida auris* was detected as a causative pathogen. 92% carbapenem resistance and 7% colistin resistance were determined in *A. baumannii*, the most common HAI pathogen. In *K. pneumoniae*, 58% carbapenem resistance and 26% colistin resistance were detected. Oxacillin resistance was 71% in coagulase negative staphylococci (CNS), and 43% in *Staphylococcus aureus*. Teicoplanin and vancomycin resistance in *E. faecium* were 19% and 14%, respectively.

### HAIs in neonatal ICUs

In 47 HCFs applying surveillance, 1237 HAIs were found in a total of 51,003 patients. The most common DA-HAI was CLABSI, while the most common NDA-HAIs were BSIs and urinary tract infections (UTI). *K. pneumoniae* (22.4%), *A. baumannii* (9.5%), and CNS (8.6%) were the most commonly isolated pathogens. 39% carbapenem resistance and 36% colistin resistance were detected in *K. pneumoniae*; 94% carbapenem resistance and 3% colistin resistance were detected in *A. baumannii*. Oxacillin resistance was 72% in CNSs, while it was 37% in *S. aureus*. Teicoplanin and vancomycin resistance in *E. faecium* were 38% and 43%, respectively.

### HAIs in pediatric ICUs

In 38 HCFs performing pediatric ICU surveillance, 806 HAIs were detected in a total of 16,274 patients. While the most common DA-HAI was CLABSI, the most common NDA-HAI was BSI. The three pathogens that were found to be the most common were *K. pneumoniae* (22.9%), *P. aeruginosa* (16.2%), and *A. baumannii* (12.16%). A carbapenem resistance ratio of 64% and a colistin resistance ratio of 11% were found in *K. pneumoniae*, whereas a carbapenem resistance ratio of 89% and a colistin resistance ratio of 11% were found in *A. baumannii.* The percentage of oxacillin-resistant CNS was determined to be 63 percent, whereas the percentage of resistant *S. aureus* was 44 percent. Teicoplanin and vancomycin resistance was discovered in 33% of the *E. faecium* strains identified, which was accounted three of the nine strains.

### IPC level, IPC core components, and HAIs

In adult ICUs, all infection rates were higher in HCFs that had moderate IPC levels when compared to facilities that had advanced IPC levels (Table 5). There was a trend towards lower HAI rates when the IPC level was higher, albeit one that was not statistically significant. This difference in the rate of VAP in the adult ICU was statistically significant (*p* = 0.047).

**Table 5:** The effect of IPC levels on health care-associated infection rates in ICUs

	Intermediate IPC level	Advanced IPC level	*P*
Adult ICU (n = 63)			
CLABSI rate (per 1000 central line days)	5.25 (8.52)	4.64 (6.03)	0.54
CA-UTI rate (per 1000 urinary catheter days)	1.12 (1.48)	0.81 (1.19)	0.16
VAP rate (per 1000 ventilator days)	5.42 (11.62)	0.65 (8.00)	0.047
Pediatric ICU (n = 36)			
CLABSI rate (per 1000 central line days)	5.31 (11.24)	2.33 (7.51)	0.91
CA-UTI rate (per 1000 urinary catheter days)	0.65 (2.74)	0.00 (0.40)	0.15
VAP rate (per 1000 ventilator days)	2.77 (3.58)	1.05 (3.03)	0.26
Neonatal ICU (n = 43)			
CLABSI rate (1000 per central line days)	2.30 (7.09)	1.70 (7.21)	0.73
CA-UTI rate (1000 per urinary catheter days)	0.00 (1.05)	0.00 (0.00)	0.96
VAP rate (1000 per ventilator days)	0.50 (6.63)	0.00 (2.04)	0.41

*ICU* intensive care unit, *IPC* infection prevention and control, *CLABSI* Central line- associated blood stream infections, *CA-UTI* Catheter-associated urinary tract infection, *VAP* Ventilator-associated pneumonia

In general, the rates of HAIs were higher in ICUs of hospitals that provide tertiary care (TRH, CH, and UH) as opposed to secondary care hospitals (SH and PH), and in hospitals that have a bed capacity of more than 1000 as opposed to hospitals that have a bed capacity of less than 500. The presence of a certified infection control doctor and/or nurse, hand hygiene observation, and antiseptic use feedback should be done monthly rather than for periods longer than three months, and the number of nurses per bed in the day shift is greater than 0.5, are all factors that have been linked to a reduction in HAI rates, though this varies depending on the type of ICU and developing HAI (Table 6, Additional File 4). Particularly in adult ICUs of HCFs with a hospital bed capacity of more than 500 patients, there were fewer than 0.5 nurses per bed for ICUs during a day shift, which significantly raised the rate of VAE (*p* = 0.015).

**Table 6:** Certain IPC components are linked to health care-associated infections in ICUs

	VAP in ICUs			CLABSI in ICUs			CA-UTI in ICUs			Non-Invasive HAI in ICUs		
	Neonatal Median	Pediatric Median	Adult Median	Neonatal Median	Pediatric Median	Adult Median	Neonatal Median	Pediatric Median	Adult Median	Neonatal Median	Pediatric Median	Adult Median
**Hospital**												
TRH	0.60	1.91	0.27	4.83	1.27	4.80*	0.00	0.00	0.68	0.60	0.11*	1.16
UH	0.00	1.19	5.61	2.37	2.02	5.80*	0.00	0.00	1.24	1.53	1.60*	1.49
CH	6.37	0.76	0.80	6.69	8.20	4.58*	0.00	0.26	0.82	0.97	1.23	0.85
SH	0.00	-	8.63	1.22	-	1.86	0.00	-	1.02	0.00	-	2.23
PH	0.00	-	0.56	0.68	-	0.00	0.00	-	1.22	2.20	-	3.63
**Cerficated infection control doctor**												
Yes	0.00	1.61	1.02*	1.98	3.23	4.81	0.00*	0.00	0.90	0.79	0.80	1.20
No	0.00	1.48	12.91*	5.06	0.29	4.39	45.04*	0.26	2.15	2.07	2.81	3.35
**Cerficated infection control nurse**												
Yes		1.24	1.34	2.52	3.23	4.78	0.00	0.00	0.85*	0.80	1.14	1.35
No	0.00	1.48	9.07	0.88	0.29	12.2	0.00	0.00	3.25*	1.1	0.13	2.6
**Feedback for hand hygiene compliance frequency**												
Once a month	0.00	0.00*	2.91	0.44	0.59	1.28	0.00	0.00	1.14	0.18	0.68	3.66
Once in three months	0.21	1.24	1.18	2.43	2.35	5.27	0.00	0.00	0.89	0.80	1.01	1.27
Less than once in three months	1.95	4.45*	9.64	7.43	8.35	6.34	-	4.04*	1.15	1.12	7.33	5.39
None	-	-	1.48	-	-	2.79	-	-	0.42	-	-	0.91
**Frequency of report of antiseptic using**												
Once a month	0.00	0.00	0.54	0.88	0.59	0.00*	0.00	0.00	0.23	1.99	0.26	0.45
Once in three months	0.12	2.21	1.01	1.70	2.35	4.81*	0.00	0.00	0.90	0.65	0.88	1.62
None	0.00	0.48	6.75	4.37	7.47	5.04*	0.00	0.79	1.06	1.95	1.36	1.19
**Nurse per bed in the daytime**												
< 0.5	0.00	3.50	6.75	2.37	10.5	4.68	0.00	0.00	0.81	0.00	1.30	1.62
≥ 0.5	0.58	1.50	0.71	1.82	2.00	4.80	0.00	0.00	1.01	0.83	0.65	1.27
**Nurse per bed at night**												
< 0.5	0.00	2.00	5.56	2.49	6.00	4.68	0.00	1.00	1.09	1.09	1.28	1.64
≥ 0.5	0.00	1.50	1.01	1.21	4.50	4.80	0.00	0.00	0.83	0.60	0.80	1.28
**Number of hospital bed**												
≤ 500	0.00	0.00	0.56	0.00	0.50*	2.17*	0.00	0.00*	0.60	0.19	0.34	1.06
501—1000	0.12	1.50	0.74	3.74	4.50	5.12	0.00	0.00	0.96	0.60	1.60	1.25
> 1001	3.39	4.00	6.06	3.54	10.0*	5.87*	0.00	0.50*	1.08	1.06	0.77	1.93

*VAP* Ventilator-associated pneumonia, *CLABSI* Central line- associated blood stream infections, *CA-UTI* Catheter-associated urinary tract infection, *HAI* Health care-associated infection*, IPC* Infection prevention and control, *TRH* Training and Research Hospitals, *UH* University Hospitals, *CH* City Hospitals, *SH* State Hospitals, *PH* Private Hospitals, *ICU* Intensive care unit, * Significant relationship between HAIs and IPC components (p < 0.005)

## Discussion

To our knowledge, this is the first comprehensive national study to evaluate IPC practices in HCFs in Türkiye, a middle-income developing country, using the IPCAF questionnaire. In addition, the respective effects of IPC levels (IPCAF score) and the implementation of CCs on the rates of HAI developing in the ICU were investigated. In our study, in the evaluation made in terms of core components after the national infection control programme had been implemented in Türkiye for 15 years, it was determined that the application rates of IPC CCS in health institutions were quite high. Although performance observation was not made for IPC practices in the study, as a result of the evaluation made using IPCAF, the opportunity to define the strengths, gaps, and key points for improvement in IPC practices in our country was obtained. The results we obtained from 68 hospitals are acceptable for the whole country due to the participation in the study of seven regions of Türkiye and the regulation of infection prevention and control, which has been well established in the country for 15 years.

When all the HCFs in our study were evaluated, the participating HCFs (75%) had an advanced level of IPC (668.8 points) based on CCs. Internationally, in a recent WHO global study that analyzed 81 nations, advanced levels of IPC (IPCAF median score of 605 points) were predominantly found in high-income countries [26]. In contrast, lower scores were reported for low-income countries (385 points), LMIC (504 points), and public facilities (515 points) [26]. It has been reported that 84.5% of hospitals in Germany have advanced level of IPC [27]. Similarly, advanced IPC levels were detected in Austria [28]. On the other hand, it was demonstrated that all of the institutions evaluated in Islamabad had IPCAF scores below 200 [29]. In another study, only 14.3% of institutions in Ghana, a country with limited resources, had advanced IPC levels. [30].

According to the findings of our research, despite the fact that our nation is classified as a middle-income developing country, it has a high IPCAF score that is comparable to that of high-income countries. However, the fact that some hospitals are only at the basic or intermediate levels of the IPC demonstrates that there is still opportunity for progress in this subject. In addition, the statement that 100% of PHs had advanced IPC levels may be misleading when making generalizations due to the fact that our study only included three PHs.

The highest IPC CC scores reported in HCFs around the worldwere in CC8 (built environment) and CC2 (guidelines) [26]. In contrast, CC7 (workload, staffing, and bed occupancy), CC5 (multimodal strategies), and CC3 (IPC education and training) were the lowest-rated CCs [26–28]. On the other hand, CC4 (HAI surveillance) and CC6 (monitoring IPC implementation and feedback) were reported as the lowest-scoring CCs in low-income countries [26]. Even in high-income countries such as Germany and Austria, workloads, implementation of multimodal strategies, and a lack of feedback have been reported [27, 28]. In our research, the highest scoring component was CC2 (IPC guidelines; median 98.8 points), while the lowest-scoring components were CC7 (workload, staff, and bed occupancy; median 70.0 points) and CC5 (multimodal strategies; median 75.0 points).

According to the results of our research, the IPC programmes are generally well known in Türkiye, as indicated by the median score of 90 points on the IPCAF CC1 assessment. One possible explanation for this is that the government has strict regulations for controlling infectious diseases. For all HCFs, a score of 92.5 points was determined to be the median for CC4 (HAI surveillance). It demonstrates that there is a well-established and well-functioning surveillance system comparable to that of industrialized countries, as well as that monitoring and feedback of IPC procedures are effectively implemented. The poor score on the CC5 (multimodal strategies) section may be due to the fact that multimodal strategies are still a relatively novel concept in infection control. A median score of 75 points for CC5 in our analysis demonstrates that this component has much potential for development. However, it also indicates that this new multimodal strategies of doing things has been adopted by a significant number of HCFs.

In our study, it was observed that the requirement of one IPC specialist per 250 beds, as recommended by WHO, and an infection control nurse for 150 beds, as recommended by national regulations, could not be met. Furthermore, other than IPC personnel, only 45 (66.2%) of the institutions had personnel present who possessed the necessary abilities to assist in educational work. The presence of qualified personnel to assist with training in institutions alleviates the workload of an insufficient number of IPC professionals.

In addition, we detected a significant shortcoming in the study: only a relatively small number of institutions offer the integration of IPC training into clinical practice for all medical subspecialties. Due to the fact that IPC practices affect every department within the organization, additional arrangements are needed to ensure the integration of IPC training into all specialties.

Another issue that was discovered was that most HCFs did not provide patients and members of their families with access to private IPC training. There is limited data in the literature on IPC training for patients and family members. The CDC recommends that patients and family members receive education on IPC [31]. However, a systematic review highlighted that IPC education for patients is very low [32]. Especially in countries where family members participate in patient care, it is important to make arrangements for the IPC training of patient relatives [33]. It was reported that nurses who know a lot about IPC give more training to patients and their family members [34]. This means that nurses who are trained in IPC have a very important role to play in increasing the participation and education of patients and family members in IPC.

In our study, 74% of HCFs reported that IPC goals and indicators had the support of their leaders. However, just 22% of HCFs had a budget for IPC programmes. In addition, 7.3% of the HCFs reported that they lacked sufficient IT assistance to manage to monitor. There is a requirement for regulations to resolve these deficiencies, which can be remedied with the assistance of the institution's administrators.

In this study, workload and the limited number of nurses were reported as the most critical gaps in parallel with the literature. In addition to the fact that there were not enough nurses working in infection control, it was discovered that there were not enough nurses working in ICUs, which is where the majority of infections in hospitals occur. The ratio of nurses to patients in the 3rd level ICUs was higher than 0.5 in 62% of the day shift and 47% of the night shift. Although not statistically significant, the HAI rate was higher in ICUs with a nurse/patient ratio of < 0.5. Studies have reported that the risk of developing infection increases with an increase in workload, especially in critically ill patients [35]. A study showed that a low nurse/patient ratio was associated with a 50% increase in the risk of HAIs. A nurse/patient ratio of less than 1.9 was associated with an increased risk of disease [36]. In another study, it was estimated that 26.7% of all infections could be prevented if the nurse-patient ratio was kept at > 2.2 [35].

In addition, a study showed that carbapenem resistance in gram-negative bacteria increased with a decrease in nurse density [37]. This information underscores the necessity to consider nurses' contributions to the fight against AMR in future health policy and suggests that nurse competence may play a role in halting the development of AMR. The key determinant in the prevention of HAIs in critically ill patients is to maintain a higher level of nursing staff. With the help of this research, it has been understood that it is very important to take action to increase the number of nurses in our country.

ICUs are more risky areas for the development of HAIs. In particular, the majority of HAIs are associated with the invasive devices used [9]. In a study by Blot et al., the rate of VAP in 1000 ventilator days in ICU was 0.9–4.4 in the USA, 9.8 in Europe, 17.8 in China, and 17–29 in Türkiye; CLABSI per 1000 catheter days has been reported to be 0.9–3.4, 3.6, 9.7, and 12 in America, Europe, China, and Türkiye, respectively [10]. In our study, although it varies according to the type of HCFs, VAP in 1000 ventilator days was 4.8 in adult ICU, 1.9 in neonatal ICU, and 2.1 in pediatric ICU; CLABSI was found to be 7.1 in adult ICU, 6.9 in neonatal ICU, and 4.9 in pediatric ICU at 1000 catheter days. In our study, it is noteworthy that the CLABSIs were higher than those in America and Europe. A significant increase in hospital-acquired BSIs in COVID-19 and non-COVID-19 patients was found in 2020 compared to the pre-pandemic period in a group of hospitals in London [38]. The rise in BSIs identified in our investigation might be due to the COVID-19 pandemic. It has been thought that the need to employ less qualified intensive care nurses due to the increased workforce during the pandemic process may have contributed to this increase.

Research has underlined the impact of the growing usage of noninvasive high-flow ventilation on increased nonventilated hospital-acquired pneumonia (4.5 per 1000 patient days) [39]. The increasing use of noninvasive high-flow ventilation during the COVID-19 pandemic may have contributed to our research’s elevated prevalence of nosocomial pneumonia in the adult ICU.

In a study evaluating the effectiveness of a national surveillance system supported by a national IPC program (certification of IPC personnel, training of healthcare personnel, multimodal hand hygiene campaigns, guidelines, surveillance with internationally accepted standardized definitions, rapid data reporting and analysis methods to the Ministry, and continuous surveillance) to reduce DA-HAIs in Türkiye based on 1000 invasive device days from 2008 to 2017, VAE decreased from 16.69 to 4.86, CA-UTI from 4.98 to 1.59, and CLABSI from 5.65 to 2.82. When evaluated according to institutions, from 2008 to 2017, VAE in 1000 ventilator days has been reported to decrease from 22.34 to 10.7 in UHs; from 14.68 to 5.89 in TRHs; from 16.94 to 3.91 in SHs; and from 10.28 to 2.64 in PHs [23]. In our study's 2021 data, we identified a reduced incidence density of VAE and a declining trend in CA-UTI but a greater incidence density of CLABSI.

In the national 2020 surveillance report, the most often reported HAIs are BSI, pneumonia, and UTI. According to the paper, the most prevalent causes of HAIs are *Klebsiella* spp. (19.2%), *Acinetobacter* spp. (17.6%), *Pseudomonas* spp. (9.2%), and *E. coli* (8.6%). Furthermore, the most commonly discovered gram-positive pathogens were *Enterococcus* spp. (7.3%), CNS (5.3%), and *S. aureus* (4.5%) [40]. In another study conducted in our country, it was reported that the most common HAIs in ICUs were BSI, VAP, CA-UTI, CLABSI, and *A. baumannii*, *K. pneumoniae*, *Enterococcus* spp., and *P. aeruginosa* were the most common causative pathogens in these infections [41]. Although the incidence varies by facility and ICU type, the most often found HAIs in our research are BSI, pneumonia, and UTI, and the most common HAI pathogens, particularly *Acinetobacter* and *Klebsiella* species, are gram-negative bacteria.

*Candida* *parapsilosis* is a prominent cause of HAIs, particularly CLABSI [42, 43]. Similar to the literature, it was the most often isolated fungus among non-albicans *Candida* in HAIs in our investigation. Studies have shown that *C. parapsilosis* colonizes the hands of hospital staff and surfaces in hospitals [44, 45]. HAIs caused by *C. parapsilosis* have been documented as a consequence of cross-contamination with healthcare workers’ hands [46]. Similarly, environmental surfaces were reported as an important reservoir for most nosocomial pathogens, including *Acinetobacter* spp. and *Klebsiella* spp., and this contaminated hospital environment played an important role in the increase of HAIs [47]. In our study, the frequent detection of *A. baumanii* (9.5–21.2%) and *K. pneumoniae* (16.3–22.9%) suggested insufficient environmental cleaning and disinfection. It might point to the need for more frequent cleaning of hospital surfaces as well as improved hand hygiene compliance.

AMR is now one of the most serious worldwide issues, increasing health-care expenditures and leading to treatment failure and mortality. Among pathogens found in HAI in Europea, carbapenem resistance is high, particularly in *Acinetobacter* species, *K. pneumoniae*, and *P. aeruginosa* [11]. Among HAI pathogens found in European ICUs, carbapenem resistance was reported in 15.2% of *Klebsiella* spp., 25.9% of *P. aeruginosa*, 63.9% of *A. baumannii*, and 0.8% of *E. coli* [9].

Carbapenem resistance 94.5–98.3% and colistin resistance 4.5–7.3% in *Acinetobacter* spp., carbapenem resistance 59.8–75.2% and colistin resistance 30.4–36.8% in *K. pneumoniae*, carbapenem resistance 51.5–66.8% and colistin resistance 4.4%-8.4% in *P. aeruginosae* were reported, which are the causative pathogens of the most common HAIs (BSI, pneumonia, and UTI) in our country [40]. In the 2017 annual epidemiological report of the European Centre for Disease Prevention and Control (ECDC), methicillin resistance in *S. aureus* was reported at 23.5%, and glycopeptide resistance in *Enterococcus* spp. was 9.5% in ICUs [9]. Methicillin resistance in *S. aureus* and glycopeptide resistance in *Enterococcus* spp. were shown to be greater in our research. In all ICUs, methicillin resistance in the CNS exceeds 60%. This high level of antibiotic resistance shows that regulatory steps to minimize AMR are urgently needed.

## Limitations

This study has some limitations. The results we obtained from 68 hospitals are acceptable for the whole country due to the participation in our study of seven regions of Türkiye and the regulation of infection prevention and control, which has been well established in the country for 15 years. However, it is one of the limitations of our study that it constitutes approximately 4.4% of the health institutions throughout the country. Second, a self-assessment was done, and the reported data was not validated. In this study, the inability to monitor the performance of IPC applications is a limitation. Meeting official criteria (e.g., the presence of guidelines) may not necessarily reflect adherence to guidance, and the absence of formal criteria may not imply low adherence to IPC measures. Fourth, AMR data may cause variations in reported data due to changes in laboratory testing and reporting methods across centers. Fifth, although the number of centers providing data is good for country conditions, evaluations such as many stratified analyses and multivariate analyses that were considered during the statistical evaluation could not be made. Because of the reduction in the number of centers per group, the chance of type 2 errors has risen in the stratifications that may be created. Finally, the effect of the basic IPC level on HAI could not be studied because one of the two centers where a basic infection control programme was used only had an ICU for adults and the other only had an ICU for pediatric and neonatal. So, when IPCAF looked at the effect of IPC level on infection rates, the effect of HCFs with a basic IPC level couldn't be looked at.

## Conclusion

In conclusion, this is the first national study in a developing country with limited resources to analyze the IPC CCs and the association between IPC CCs and HAI rates. Even though IPC research in Türkiye began 50 years after that in the United States and Europe, significant progress has been achieved in establishing the CCs of IPC in the previous 15 years. Despite the high success rate of using the CCs of IPC in our research, HAIs in ICUs remain significant despite a reduction. HAIs are concerning because they contribute to the spread of AMR and threaten patient safety. According to the study's findings, the most pressing issue seems to be a shortage of nurses. HAIs can be significantly prevented by bringing the number of nurses to ideal ratios and reducing the workload in ICUs. In hospitals, the number of certified infection control doctors and nurses should be idealized, and their training should be updated. It is recommended to plan studies that will measure the effect of future interventions on the subject. When all ICUs for adult, pediatric, and neonatal were looked at, it was found that the most common DA-HAI was CLABSI, the most common NDA-HAI was BSI, and the most commonly isolated pathogens from HAI were gram-negative bacteria. It has been reported that resistance rates are high in these microorganisms. Therefore, the “Antimicrobial Stewardship Programme" should be launched nationally.

## Supplementary Information

**Additional file 1** Infection Prevention and Control Assessment Framework (IPCAF) Questionnaire, Turkish Version.

**Additional file 2** Results of the Infection Prevention and Control Assessment Framework in 68 participating hospitals.

**Additional file 3** The features of ICUs and HAIs in ICUs, 2021.

**Additional file 4** Certain IPC components are linked to health care-associated infections.

## Data Availability

The datasets in the study are available from the corresponding author on reasonable request.

## Abbreviations

AMR: Antimicrobial resistance
AMS: Antimicrobial stewardship
BSI: Blood stream infections
CA-UTI: Catheter-associated urinary tract infection
CC: Core component
CDC: Centers for Disease Control and Prevention
CH: City hospital
CLABSI: Central line- associated blood stream infections
CNS: Coagulase negative staphylococci
COVID-19: Coronavirus disease 2019
DA-HAI: Device- associated health care-associated infection
ECDC: European Centre for Disease Prevention and Control
EKMUD: Türkiye Infectious Diseases and Clinical Microbiology Specialization Association
HAI: Health care-associated infection
HAIs: Health care-associated infections
HCFs: Health care facilities
ICC: Infection control committee
ICU: Intensive care unit
IQR: Interquartile range
IPC: Infection prevention and control
IPCAF: Infection Prevention and Control Assessment Framework
IT: Information technology
LMICs: Low- and middle-income countries
NDA-HAI: Non-device- associated health care-associated infection
NHSN: National Healthcare Safety Network
PH: Private hospital
SD: Standard deviation
SH: State hospital
SSI: Surgical site infection
TRH: Training and research hospital
TRNC: Turkish Republic of Northern Cyprus
UH: University hospital
UTI: Urinary tract infection
VAE: Ventilator-associated event
VAP: Ventilator-associated pneumonia
WHO: World health organization
