# Supplementary material for: Evaluation of the implementation of WHO infection prevention and control core components in Turkish health care facilities: results from a WHO infection prevention and control assessment framework (IPCAF)—based survey
Source: Antimicrob Resist Infect Control. 2023 Feb 13;12:11. doi: 10.1186/s13756-023-01208-0 (PMC9923650; doi:10.1186/s13756-023-01208-0)
Supplement: Supplementary file 1 — Additional file 1 Infection Prevention and Control Assessment Framework (IPCAF) Questionnaire, Turkish Version. [file 13756_2023_1208_MOESM1_ESM.docx]

| **Additional File 1.** The Infection Prevention and Control Assessment Framework (IPCAF) Questionnaire,  Enfeksiyon Önleme ve Kontrol Değerlendirme Çerçevesi Anketi, Turkish Version | | |
| --- | --- | --- |
| **TEMEL BİLEŞEN 1: EÖK PROGRAMI** | | |
| **Soru** | **Cevap** | **Puan** |
| 1. EÖK programınız var mı? | Hayır | 0 |
|  | Evet, amaç açıkça tanımlanmamış | 5 |
|  | Evet, amaç ve yıllık aktivite planı açıkça tanımlanmış | 10 |
| 1. EÖK programı EÖK uzmanını kapsayan bir EÖK ekibiyle destekleniyor mu? | Hayır | 0 |
|  | Ekip yok, sadece bir EÖK lokal kişi | 5 |
|  | Evet | 10 |
| 1. EÖK ekibinde tam zamanlı çalışan bir EK hemşiresi veya doktoru var mı? | Bir EÖK uzmanı yok | 0 |
|  | Sadece partime çalışan bir EÖK uzmanı var | 2,5 |
|  | Evet, >250 yatak başına bir | 5 |
|  | Evet, ≤250 yatak başına bir | 10 |
| 1. EÖK ekibi veya sorumlu kişisi EÖK faaliyetleri için ayrılmış zamanı var mı? | Hayır | 0 |
|  | Evet | 10 |
| 1. EÖK ekibinde hem hemşire hem doktor var mı? | Hayır | 0 |
|  | Evet | 10 |
| 1. EÖK ekibini aktif olarak destekleyen bir EKK var mı? | Hayır | 0 |
|  | Evet | 10 |
| 1. EKK aşağıda gösterilen grupları içeriyor mu? | | |
| Kıdemli kurum lideri (idari müdür, şef, tıbbi direktör, yönetici) | Hayır | 0 |
|  | Evet | 5 |
| Kıdemli klinik personeli (Ör; doktor, hemşire) | Hayır | 0 |
|  | Evet | 2,5 |
| Kurum yönetimi (örneğin, su, sanitasyon ve hijyen konularına yönelik biyogüvenlik, atık için görevlendirilenler) | Hayır | 0 |
|  | Evet | 2,5 |
| 1. EÖK hedefleri açıkça tanımlanmış mı (spesifik kritik alanlarda)? | Hayır | 0 |
|  | Evet, sadece EÖK amaçları | 2,5 |
|  | Evet, EÖK hedefleri ve ölçülebilir sonuç göstergeleri (yani iyileştirme için yeterli önlemler) | 5 |
|  | Evet, EÖK hedefleri ve ölçülebilir sonuç göstergeleri ve gelecek hedeflerin belirlenmesi | 10 |
| 1. Üst düzey kurum lideri EÖK programı için açık bir taahhüt ve destek gösteriyor mu? | | |
| Özellikle EÖK programı için tahsis edilen bir bütçeyle (maaşlar dahil EÖK faaliyetlerini kapsayan) | Hayır | 0 |
|  | Evet | 5 |
| Kurum içinde EÖK hedefleri ve göstergeleri için kanıtlanabilir destekle (ör; yönetici düzeyindeki toplantılarda, yönetici toplantılarında, morbidite ve mortalite toplantılarına katılım)? | Hayır | 0 |
|  | Evet | 5 |
| 1. Kurumun rutin günlük kullanım için mikrobiyoloji labortuvar desteği var mı (kurum içi veya dışında) | Hayır | 0 |
|  | Evet, ancak sonuçları güvenilir değil (zamanında ve yeterli kalite açısından) | 5 |
|  | Evet, sonuçları güvenilir (zamanında ve yeterli kalite açısından) | 10 |
| **Ara toplam puan** | **/100** | |

| **TEMEL BİLEŞEN 2**: EÖK KLAVUZLARI | | |  |  |
| --- | --- | --- | --- | --- |
| **Soru** | | | **Cevap** | **Puan** |
| 1. Kurumunuz klavuz geliştirmek veya uyarlamak için uzmanlığa (EÖK ve/veya enfeksiyon hastalıkları) sahip mi? | | | Hayır | 0 |
|  |  |  | Evet | 7,5 |
| 1. Kurumunuzda aşağıdaki klavuzlar mevcut mu? | | | | |
| Standart önlemler? | | | Hayır | 0 |
|  |  |  | Evet | 2,5 |
| El hijyeni? | | | Hayır | 0 |
|  |  |  | Evet | 2,5 |
| Bulaş yolu önlemleri? | | | Hayır | 0 |
|  |  |  | Evet | 2,5 |
| Salgın hazırlığı ve yönetimi? | | | Hayır | 0 |
|  |  |  | Evet | 2,5 |
| CAE önleme? | | | Hayır | 0 |
|  |  |  | Evet | 2,5 |
| Damar içi kateter enfeksiyonlarını önleme? | | | Hayır | 0 |
|  |  |  | Evet | 2,5 |
| Hastane kökenli pnömoniyi önleme? | | | Hayır | 0 |
|  |  |  | Evet | 2,5 |
| Kateter ilişkili üriner sistem enfeksiyonlarını önleme? | | | Hayır | 0 |
|  |  |  | Evet | 2,5 |
| Çok dirençli mikroorganizmaların yayılımını önleme? | | | Hayır | 0 |
|  |  |  | Evet | 2,5 |
| Dezenfeksiyon ve sterilizasyon? | | | Hayır | 0 |
|  |  |  | Evet | 2,5 |
| Sağlık çalışanlarının sağlığının korunması? | | | Hayır | 0 |
|  |  |  | Evet | 2,5 |
| Enjeksiyon güvenliği? | | | Hayır | 0 |
|  |  |  | Evet | 2,5 |
| Atık yönetimi | | | Hayır | 0 |
|  |  |  | Evet | 2,5 |
| Antibiyotik yönetimi | | | Hayır | 0 |
|  |  |  | Evet | 2,5 |
| 1. Kurumunuzdaki klavuzlar ulusa/uluslararası klavuzlarla (varsa) tutarlı mı? | | | Hayır | 0 |
|  |  |  | Evet | 10 |
| 1. Temel EÖK standartları yönetilirken lokal gereksinim ve kaynaklara göre klavuzların uygulanması uyarlanıyor mu? | | | Hayır | 0 |
|  |  |  | Evet | 10 |
| 1. EÖK personeline ek olarak ön saflardaki sağlık çalışanları EÖK klavuzlarının uygulanmasının hem planlaması hem de yürütülmesinde yer alıyor mu? | | | Hayır | 0 |
|  |  |  | Evet | 10 |
| 1. EÖK personeline ek olarak ilgili paydaşlar (Ör; lider doktorlar ve hemşireler, hastane yöneticileri, kalite yönetimi) EÖK klavuzlarının geliştirilmesi ve uyarlanmasına dahil mi? | | | Hayır | 0 |
|  |  |  | Evet | 7,5 |
| 1. Sağlık çalışanları, kurumda yeni veya güncellenen EÖK klavuzlarına ilişkin spesifik eğitim alıyor mu? | | | Hayır | 0 |
|  |  |  | Evet | 10 |
| 1. Kurumunuzda en azından bazı EÖK yönergelerinin uygulanmasını düzenli olarak izliyor musunuz? | | | Hayır | 0 |
|  |  |  | Evet | 10 |
| **Ara toplam puan** | **/100** | | | |
| **TEMEL BİLEŞEN 3**: EÖK EĞİTİM VE ÖĞRETİM | | | | |
| **Soru** | | **Cevap** | | **Puan** |
| 1. EÖK eğitimine liderlik etmek için EÖK uzmanlığına sahip (EÖK ve/veya enfeksiyon hast) personel var mı? | | Hayır | | 0 |
|  |  | Evet | | 10 |
| 1. Ek olarak EÖK personeli dışında eğitmen ve mentor olarak hizmet etmek için yeterli becerilere sahip personel var mı? (Ör, ilişki kuran hemşireler ve doktorlar, şampiyonlar) | | Hayır | | 0 |
|  |  | Evet | | 10 |
| 1. Kurumunuzda sağlık çalışanları EÖK konusunda ne sıklıkla eğitim alıyor? | | Hiç veya nadiren | | 0 |
|  |  | Sadece yeni başlayan sağlık çalışanları için oryantasyon | | 5 |
|  |  | Yeni başlayan sağlık çalışanları için oryantasyon ve sağlık çalışanları için düzenli (en az yılda bir) sunulan ancak zorunlu olmayan EÖK eğitimi | | 10 |
|  |  | Yeni başlayan sağlık çalışanları için oryantasyon ve tüm sağlık çalışanları için düzenli (en az yılda bir) sunulan ancak zorunlu EÖK eğitimi | | 15 |
| 1. Kurumunuzda hasta bakımı ile doğrudan ilgilenen temizlik ve diğer personel EÖK konusunda ne sıklıkla eğitim alıyor? | | Hiç veya nadiren | | 0 |
|  |  | Sadece yeni başlayan diğer personel için oryantasyon | | 5 |
|  |  | Yeni başlayan diğer personel için oryantasyon ve diğer personel için düzenli (en az yılda bir) sunulan ancak zorunlu olmayan EÖK eğitimi | | 10 |
|  |  | Yeni başlayan diğer personel için oryantasyon ve diğer personel için düzenli (en az yılda bir) sunulan ancak zorunlu EÖK eğitimi | | 15 |
| 1. Kurumunuzda yönetici ve idari personel EÖK ile ilgili genel eğitim alıyor mu? | | Hayır | | 0 |
|  |  | Evet | | 5 |
| 1. Sağlık çalışanları ve diğer personel nasıl eğitiliyor? | | Mevcut eğitim yok | | 0 |
|  |  | Sadece yazılı bilgi ve/veya sözlü talimat ve/veya e-öğrenme kullanma | | 5 |
|  |  | Ek interaktif eğitim oturumlarını içerir (Ör; simülasyon ve/veya yatak başı eğitimi) | | 10 |
| 1. Eğitim programlarının etkinliği periyodik değerlendiriliyor mu? | | Hayır | | 0 |
|  |  | Evet, fakat düzenli değil | | 5 |
|  |  | Evet, düzenli (en az yıllık) | | 10 |
| 1. EÖK eğitimi, klinik uygulama ve diğer uzmanlık eğitimi ile entegre mi? (Ör, EÖK’nün özelliklerini içeren cerrahların eğitimi) | | Hayır | | 0 |
|  |  | Evet, bazı disiplinlerde/bilim dalı | |  |
|  |  | Evet, tüm disiplinlerde | | 10 |
| 1. Potansiyel SHİE en aza indirmek için hastalar veya aile üyeleri için özel EÖK eğitimi var mı? (Ör, bağışıklığı baskılanmış hastalar, invaziv cihazı olan hastalar, çok ilaca direçli enfeksiyonu olan hastalar) | | Hayır | | 0 |
|  |  | Evet | | 10 |
| 1. EÖK personeli için sürekli geliştirme/eğitim sunuluyor mu? (Ör, düzenli olarak konferanslara, kurslara katılarak) | | Hayır | | 0 |
|  |  | Evet | | 10 |
| **Ara toplam puan** | | **/100** | | |

| **TEMEL BİLEŞEN 4**: SBİE SÜRVEYANSI | | |
| --- | --- | --- |
| **Soru** | **Cevap** | **Puan** |
| Sürveyansın organizasyonu | | |
| 1. Sürveyans, EÖK programınızın tanımlanmış bir bileşeni midir? | Hayır | 0 |
|  | Evet | 5 |
| 1. Sürveyans faaliyetlerinden sorumlu personeliniz var mı? | Hayır | 0 |
|  | Evet | 5 |
| 1. Sürveyans faaliyetlerinden sorumlu profesyoneller temel epidemiyoloji, sürveyans ve EÖK (sürveyans yöntemlerini, veri yönetimini ve yorumunu denetleme kapasitesi) konusunda eğitim almışlar mı? | Hayır | 0 |
|  | Evet | 5 |
| 1. Sürveyansınızı yönetmek için bilişim/IT desteğiniz var mı? (Ör, ekipman, mobil teknolojiler, elektronik sağlık kayıtları)) | Hayır | 0 |
|  | Evet | 5 |
| Sürveyans için öncelikler – bakım kapsamına göre tanımlanmış | | |
| 1. Lokal içeriğe göre sürveyans için hedef SBİE’leri belirlemek için bir önceliklendirme çalışması yapıyormusunuz? (Yani kurum morbidite ve mortalitesi) | Hayır | 0 |
|  | Evet | 5 |
| 1. Kurumunuzda aşağıdakiler için sürveyans yapılmaktadır: | | |
| CAE | Hayır | 0 |
|  | Evet | 2,5 |
| İnvaziv araç ilişkili enf (Ör, Kİ-ÜSE, SKİ-KDE, VİP) | Hayır | 0 |
|  | Evet | 2,5 |
| Klinik tanımlı enfeksiyonlar (Ör, mikrobiyolojik test yokluğunda klinik semptom ve bulgular baz alınarak tanımlanmış) | Hayır | 0 |
|  | Evet | 2,5 |
| Lokal epidemiyolojik durumunuza göre çok ilaca dirençli patojenlerin neden olduğu kolonizasyon veya enfeksiyonlar | Hayır | 0 |
|  | Evet | 2,5 |
| Lokal öncelikli salgın eğilimli enfeksiyonlar (Ör, norovirus, influenza, tüberküloz, SARS, Ebola, Lassa ateşi)? | Hayır | 0 |
|  | Evet | 2,5 |
| Hassas popülasyonlardaki enfeksiyonlar (Ör, YD, YBÜ, bağışıklığı baskılanmış, yanık)? | Hayır | 0 |
|  | Evet | 2,5 |
| Klinik laboratuvar veya diğer alanlardaki enfeksiyondan etkilenebilcek sağlık çalışanları (Ör, Hepatit B veya C, HIV, influenza)? | Hayır | 0 |
|  | Evet | 2,5 |
| 1. Sürveyansınızın kurumunuzun mevcut ihtiyaçları ve öncelikleri ile uyumlu olup olmadığını düzenli olarak değerlendiriyor musunuz? | Hayır | 0 |
|  | Evet | 5 |
| Sürveyans metodu |  |  |
| 1. Güvenilir sürveyans vaka tanımları kullanıyor musunuz? (Ör, CDC NHSN/ECDC) veya delil bazlı adaptasyon proçesleri ve uzman kosultasyonları ile adapta edilmiş? | Hayır | 0 |
|  | Evet | 5 |
| 1. Uluslararası sürveyans protokollerine göre standardize veri toplama yöntemi (Ör, aktif prospektif sürveyans) kullanıyor musunuz? (Ör, CDC NHSN/ECDC) veya delil bazlı adaptasyon proçesleri ve uzman kosultasyonları ile adapta edilmiş? | Hayır | 0 |
|  | Evet | 5 |
| 1. Veri kalitesini düzenli olarak gözden geçirmek için uyguladığınız süreçler var mı? (Ör, vaka rapor formlarının değerlendirilmesi, mikrobiyoloji sonuçlarının gözden geçirilmesi, payda tespiti vb)? | Hayır | 0 |
|  | Evet | 5 |
| 1. Sürveyansınızı destekleyen yeterli mikrobiyoloji ve laboratuvar kapasitesine sahip misiniz? | Hayır | 0 |
|  | Evet, gram pozitif-negatif ayırabiliyor ancak patojen identifiye edemiyor | 2,5 |
|  | Evet, patojenleri zamanında güvenilir bir şekilde tanımlayabilir (Ör, izolatı tanımlama) | 5 |
|  | Evet, patojenleri ve antimikrobiyal direnci zamanında güvenilir bir şekilde tanımlayabilir | 10 |
| Bilgi analizi ve yayma/veri kullanımı, bağlantı ve yönetişim | | |
| 1. Sürveyans verileri, EÖK programlarının iyileştirilmesi için özel olarak hazırlanmış birim/kurum bazlı planlar yapmak için kullanılıyor mu? | Hayır | 0 |
|  | Evet | 5 |
| 1. Antimikrobiyal ilaç direncini düzenli olarak analiz ediyor musunuz? (Ör, üç ayda bir, altı ayda bir, yılda bir) | Hayır | 0 |
|  | Evet | 5 |
| 1. Düzenli olarak sürveyans bilgilerinin geri bildiriminin yapılması (Ör, üç ayda bir, altı ayda bir, yılda bir): | | |
| Ön saflardaki sağlık çalışanları (doktorlar/hemşireler)? | Hayır | 0 |
|  | Evet | 2,5 |
| Departmanın klinik şefleri/liderleri | Hayır | 0 |
|  | Evet | 2,5 |
| EKK | Hayır | 0 |
|  | Evet | 2,5 |
| Klinik dışı yönetim/yönetim (baş yönetici, baş finans görevlisi) | Hayır | 0 |
|  | Evet | 2,5 |
| 1. Sürveyans verilerinin geri bildirimini nasıl yapıyorsunuz (en az yıllık) | Yapılmıyor | 0 |
|  | Sadece yazılı/sözel bilgilendirme | 2,5 |
|  | Sunum ve interaktif problem odaklı çözüm bulma | 7,5 |
| **Ara toplam puan** | **/100** | |

| **TEMEL BİLEŞEN 5**: MULTİMODAL STRATEJİLER | | |
| --- | --- | --- |
| **Soru** | **Cevap** | **Puan** |
| 1. EÖK’de multimodal stratejiler kullanıyor musunuz? | Hayır | 0 |
|  | Evet | 15 |
| 1. Takip eden elementlerin herhangi biri veya tümünü içeren multimodal stratejilerin kullanımı: (her biri için en kesin cevabı seçin) | **Sistem değişikliği** | |
|  | Multimodal stratejileri içeren element yok | 0 |
|  | Gerekli alt yapının ve sarf malzemelerin yerinde sürekli mevcudiyetinin sağlanmasına yönelik müdahaleler | 5 |
|  | Gerekli alt yapının ve sarf malzemelerin yerinde sürekli mevcudiyetinin sağlanmasına yönelik müdahaleler ve ergonomi ve erişebilirliğin ele alınması, SVK seti ve tepsisinin en iyi yerleşimi gibi | 10 |
|  | **Eğitim ve öğretim** | |
|  | Multimodal stratejileri içermiyor | 0 |
|  | Sadece yazılı bilgi ve/veya sözlü talimat ve/veya e-öğrenme | 5 |
|  | Ek olarak interaktif eğitim oturumları (simülasyon ve/veya yatak başı eğitimi içeren) | 10 |
|  | **İzlem ve geri bildirim** | |
|  | Multimodal stratejileri içermiyor | 0 |
|  | Süreç ve sonuç göstergelerine uygunluğun izlenmesi (Ör, el hijyeni ve kateter uygulamalarının denetimleri) | 5 |
|  | Uyumun izlenmesi ve sağlık çalışanlarına ve kilit oyunculara izleme sonuçlarının zamanında geri bildiriminin sağlanması | 10 |
|  | **İletişim ve hatırlatıcılar** | |
|  | Multimodal stratejileri içermiyor | 0 |
|  | Uygulamaları teşvik etmek için hatırlatıcılar, posterler veya diğer farkındalık artırma araçları | 5 |
|  | Birimler ve disiplinler arasında ekip iletişimini geliştirmek için ek yöntemler/girişimler (örneğin, düzenli vaka konferansları ve geri bildirim turları) | 10 |
|  | **Güvenli iklim ve kültür değişikliği** | |
|  | Multimodal stratejileri içermiyor | 0 |
|  | Yöneticiler/liderler görünür bir destek gösterirler ve şampiyonlar ve rol modeller olarak hareket ederler, uyarlanabilir bir yaklaşımı teşvik ederler ve EÖK, hasta güvenliği ve kaliteyi destekleyen bir kültürü güçlendirirler | 5 |
|  | Ek olarak, ekipler ve bireyler, uygulamanın sahipliğini algılamaları için yetkilendirilir (Ör, katılımcı geri bildirim turları ile) | 10 |
| 1. EÖK multimodal stratejilerini uygulamada multidisipliner bir ekip kullanılıyor mu? | Hayır | 0 |
|  | Evet | 15 |
| 1. EÖK multimodal stratejilerini geliştirmek ve teşvik etmek için kalite iyleştirme ve hasta güvenliğinden meslektaşlarınızla düzenli olarak bağlantı kuruyor musunuz? | Hayır | 0 |
|  | Evet | 10 |
| 1. Bu stratejiler demetler veya kontrol listeleri içeriyor mu? | Hayır | 0 |
|  | Evet | 10 |
| **Ara toplam puan** | **/100** | |

| **TEMEL BİLEŞEN 6**: EÖK UYGULAMALARININ VE GERİ BİLDİRİMİN İZLENMESİ/DENETLENMESİ | | |
| --- | --- | --- |
| **Soru** | **Cevap** | **Puan** |
| 1. EÖK uygulamalarının izlenmesi/denetiminden ve geri bildiriminden sorumlu eğitimli personeliniz var mı? | Hayır | 0 |
|  | Evet | 10 |
| 1. Açık hedefler, hedefler ve faaliyetleri içeren (sistematik bir şekilde veri toplama araçları dahil) iyi tanımlanmış bir izleme planınız var mı? | Hayır | 0 |
|  | Evet | 7,5 |
| 1. Kurumunuzda hangi süreçleri ve göstergeleri izliyorsunuz? | Hiçbiri | 0 |
|  | El hijyeni uyumu (DSÖ el hijyeni gözlem aracını veya eşdeğerini kullanarak) | 5 |
|  | Damar içi katater yerleştirme ve/veya bakımı | 5 |
|  | Yara pansuman değişikliği | 5 |
|  | ÇİD mikroorganizmaların bulaşını önlemek için bulaş yolu ve izolasyon önlemleri | 5 |
|  | Klinik ortamının temizliği | 5 |
|  | Tıbbi ekipman/aletlerin dezenfeksiyonu ve sterilizasyonu | 5 |
|  | Alkol bazlı el antiseptiği veya sabun tüketimi/kullanımı | 5 |
|  | Antimikrobiyal ajanların tüketimi/kullanımı | 5 |
|  | Atık yönetimi | 5 |
| 1. DSÖ El Hijyeni Öz-Değerlendirme Anketi ne sıklıkta yapılmaktadır   Cevabı seçin | Hiç | 0 |
|  | Periyodik olarak, fakat düzenli şema yok | 2,5 |
|  | En az yıllık | 5 |
| 1. EÖK faaliyetlerinin/performansının durumu hakkında denetim raporlarının (Ör, el hijyeni uygunluk verileri veya diğer süreçlere ilişkin geri bildirim) geri bildiriminde bulunuyor musunuz?   Tümünü cevaplayın | Bildirim yok | 0 |
|  | Evet, EÖK ekibi içinde | 2,5 |
|  | Evet, denetlenen alanlardaki departman liderlerine ve yöneticilere | 2,5 |
|  | Evet, ön safhadaki sağlık çalışanlarına | 2,5 |
|  | Evet, EÖK komitesine veya bakım kalitesi komitelerine veya eşdeğerine | 2,5 |
|  | Evet, hastane yönetimine ve üst yönetime | 2,5 |
| 1. İzleme verilerinin raporlanması düzenli olarak (en az yıllık olarak) yapılıyor mu? | Hayır | 0 |
|  | Evet | 10 |
| 1. EÖK süreçleri ve göstergelerinin izlenmesi ve geri bildirimi, iyileştirme ve davranış değişikliğine yönelik ‘’suçsuz’’ bir kurumsal kültürde mi gerçekleştiriliyor? | Hayır | 0 |
|  | Evet | 5 |
| 1. Kurumunuzdaki güvenlik kültürel faktörlerini değerlendiriyor musunuz? (Ör, HSOPSC, SAQ, PSCHO, HSC22 gibi anketleri kullanarak) | Hayır | 0 |
|  | Evet | 5 |
| **Ara toplam puan** | **/100** | |

| **TEMEL BİLEŞEN 7**: İŞ YÜKÜ, PERSONEL VE YATAK DOLULUĞU | | |
| --- | --- | --- |
| Soru | Cevap | Puan |
| **Personel** | | |
| 1. Kurumunuzdaki uygun personel seviyeleri, ulusal standartlar veya DSÖ personel ihtiyacı iş yükü göstergeleri yöntemi gibi standart bir personel ihtiyacı değerlendirme amacı kullanılarak hasta iş yüküne göre değerlendiriliyor mu? | Hayır | 0 |
|  | Evet | 5 |
| 1. Kurumunuzda sağlık çalışanlarının hastalara oranı üzerinde anlaşmaya varılmış (DSÖ veya ulusal) bir oran korunuyor mu? | Hayır | 0 |
|  | Evet, birimlerin %50’sinden daha azındaki personel için | 5 |
|  | Evet, birimlerin %50’sinden daha fazlasındaki personel için | 10 |
|  | Evet, kurumdaki tüm sağlık çalışanları için | 15 |
| 1. Kurumunuzda personel seviyelerinin çok düşük olduğu düşünüldüğünde personel ihtiyaç değerlendirmelerinin sonuçlarına göre hareket edecek bir sistem var mı? | Hayır | 0 |
|  | Evet | 10 |
| **Yatak doluluğu** | | |
| 1. Kurumunuzdaki kliniklerin tasarımı, yatak kapasitesi açısından uluslararası standartlara uygun mu? | Hayır | 0 |
|  | Evet, fakat sadece belli departmanlarda | 5 |
|  | Evet, tüm departmanlarda (acil ve pediatri departmanlarını içeren) | 15 |
| 1. Kurumunuzdaki yatak doluluğu yatak başına bir hasta olarak mı tutuluyor? | Hayır | 0 |
|  | Evet, fakat sadece belli departmanlarda | 5 |
|  | Evet, tüm ünitelerde (acil ve pediatri departmanlarını içeren) | 15 |
| 1. Kurumunuzdaki hastalar, odanın dışında koridorda duran yataklara yerleştiriliyor mu (acil servisteki yataklar dahil) | Evet, haftada iki kereden daha sık | 0 |
|  | Evet, haftada iki kereden daha az sıklıkla | 5 |
|  | Hayır | 15 |
| 1. Kurumunuzda hasta yatakları arasında >1 metrelik yeterli boşluk sağlanıyor mu? | Hayır | 0 |
|  | Evet, fakat sadece belli departmanlarda | 5 |
|  | Evet, tüm departmanlarda (acil ve pediatri departmanlarını içeren) | 15 |
| 1. Kurumunuzda yeterli yatak kapasitesi aşıldığında değerlendirecek ve müdahale edecek bir sistem mevcut mu? | Hayır | 0 |
|  | Evet, bölüm başkanının sorumluluğundadır | 5 |
|  | Evet, hastane yönetiminin sorumluluğundadır | 10 |
| **Ara toplam puan** | **/100** | |

| **TEMEL BİLEŞEN 8:** KURUM SEVİYESİNDE EÖK İÇİN YAPILI ÇEVRE, MATERYAL VE EKİPMAN | | |
| --- | --- | --- |
| Soru | Cevap | Puan |
| **Su** | | |
| 1. Su hizmetleri her zaman ve tüm kullanımlar için yeterli miktarda mevcut mudur (örneğin, el yıkama, içme, kişisel hijyen, tıbbi faaliyetler, sterilizasyon, dekontaminasyon, temizlik ve   çamaşır)? | Hayır, haftada ortalama < 5 gün kullanılabilir | 0 |
|  | Evet, haftada ortalama ≥ 5 gün veya her gün mevcut ancak yeterli miktarda değil | 2,5 |
|  | Evet, her gün ve yeterli miktarda | 7,5 |
| 1. Personel, hastalar ve aileleri için her zaman ve her yerde/koğuşta güvenilir ve güvenli bir içme suyu istasyonu mevcut ve erişilebilir mi? | Hayır, mevcut değil | 0 |
|  | Bazen veya yalnızca bazı yerlerde veya tüm kullanıcılar için mevcut değildir | 2,5 |
|  | Evet, her zaman ve tüm servisler/gruplar için erişilebilir | 7,5 |
| **El hijyeni ve sanitasyon tesisleri** | | |
| 1. Tüm bakım noktalarında işlevsel el hijyeni istasyonları (yani, alkol bazlı el ovucu solüsyonu veya sabun ve su ve temiz tek kullanımlık havlular) mevcut mu? | Hayır, mevcut değil | 0 |
|  | Evet, istasyonlar mevcut, ancak sarf malzemeleri güvenilir bir şekilde mevcut değil | 2,5 |
|  | Evet, güvenilir malzemelerle | 7,5 |
| 1. Kurumunuzda ayaktan tedaviler için ≥ 4 tuvalet veya iyileştirilmiş tuvaletler veya yatan hasta alanları için her 20 kullanıcı için ≥ 1 tuvalet var mı? | Gerekli sayıdan daha az sayıda tuvalet veya hela mevcut ve çalışıyor | 0 |
|  | Yeterli sayıda mevcut ancak tümü çalışmıyor | 2,5 |
|  | Yeterli sayıda mevcut ve çalışıyor | 7,5 |
| **Güç kaynağı, havalandırma ve temizlik** | | |
| 1. Sağlık kurumunuzda, tüm kullanımlar için gece ve gündüz yeterli enerji/güç kaynağı mevcut mu? (örneğin, pompalama ve kaynar su, sterilizasyon ve dekontaminasyon, yakma veya alternatif arıtma teknolojileri, elektronik tıbbi cihazlar, sağlık işlemlerinin yapıldığı alanların genel aydınlatması sağlık hizmetlerinin güvenli bir şekilde sağlanmasını ve tuvalet ve duşların aydınlatılmasını sağlamak için) | Hayır | 0 |
|  | Evet, bazen veya yalnızca belirtilen alanlardan bazılarında | 2,5 |
|  | Evet, her zaman ve belirtilen tüm alanlarda | 5 |
| 1. Hasta bakım alanlarında işleyen çevresel havalandırma (doğal veya mekanik) var mı? | Hayır | 0 |
|  | Evet | 5 |
| 1. Zeminler ve yatay çalışma yüzeyleri için temizlik görevlileri tarafından her gün imzalanmış erişilebilir bir temizlik kaydı var mı? | Zemin ve yüzeylerin temizlendiğine dair kayıt yok | 0 |
|  | Kayıt var, ancak günlük olarak tamamlanmamış ve imzalanmamış veya güncelliğini yitirmiş | 2,5 |
|  | Evet, kayıt günlük olarak tamamlanmış ve imzalanmış | 5 |
| 1. Temizlik için uygun ve bakımlı malzemeler (örneğin deterjan, paspas, kova vb.) mevcut mu? | Malzeme yok | 0 |
|  | Evet, mevcut ancak bakımlı değil | 2,5 |
|  | Evet, mevcut ve bakımlı | 5 |
| **Sağlık bakım alanlarında hasta yerleştirme ve kişisel koruyucu donanım (KKD)** | | |
| 1. İzolasyon için tek kişilik odalarınız veya izolasyon oda sayısı yetersizse benzer patojenlere sahip hastaları kohortlamak için odalarınız var mı? (Ör, tüberküloz, kızamık, kolera, Ebola, SARS) | Hayır | 0 |
|  | Tek kişilik oda yok bunun yerine hasta kohortuna uygun odalar mevcut | 2,5 |
|  | Evet, tek kişilik odalar mevcut | 7,5 |
| 1. KKD, tüm sağlık çalışanları için tüm kullanımlar için her zaman ve yeterli miktarda mevcut mu? | Hayır | 0 |
|  | Evet, ancak yeterli miktarlarda sürekli olarak mevcut değil | 2,5 |
|  | Evet, sürekli olarak yeterli miktarlarda mevcuttur | 7,5 |
| **Tıbbi atık yönetimi ve kanalizasyon** | | |
| 1. Tüm atık üretim noktalarına yakın bulaşıcı olmayan (genel) atıklar, bulaşıcı atıklar ve kesici delici atıklar için fonksiyonel atık toplama kaplarınız var mı? | Çöp kutusu veya ayrı kesici bertarafı yok | 0 |
|  | Ayrı çöp kutuları mevcut ancak kapaklar eksik veya 3/4'ten fazla dolu; sadece iki çöp kutusu (üç yerine); veya tüm atık üretim noktalarında olmasa da bazılarında çöp kutuları | 2,5 |
|  | Evet | 5 |
| 1. Enfeksiyöz olmayan (tehlikeli olmayan/genel atık) bertaraf etmek için işlevsel bir mezar çukuru/çitle çevrili atık dökümü veya belediye toplama alanı mevcut mu? | Çukur veya başka bir bertaraf yöntemi kullanımı yok | 0 |
|  | Kurumda çukur ancak boyutları yetersiz; çukurlar/çöplükler aşırı doldurulmuş veya çitle çevrilmemiş/kilitlenmemiş; veya düzensiz belediye atık toplama | 2,5 |
|  | Evet | 5 |
| 1. Mevcut bulaşıcı ve kesici atıkların işlenmesi için işlevsel ve yeterli kapasiteye sahip bir yakma fırını veya alternatif arıtma teknolojisi (kurum içinde veya dışında mevcut ve lisanslı bir atık yönetim servisi tarafından işletilen) mevcut mu? | Hayır, hiçbiri mevcut değil | 0 |
|  | Mevcut, ancak işlevsel değil | 1 |
|  | Evet | 5 |
| 1. Bir atık su arıtma sistemi (örneğin, fosseptik ve ardından drenaj çukuru) mevcut mu (yerinde veya dışında) ve güvenilir bir şekilde çalışıyor mu? | Hayır, mevcut değil | 0 |
|  | Evet, ancak güvenilir şekilde çalışmıyor | 2,5 |
|  | Evet, güvenilir şekilde çalışıyor | 5 |
| **Dekontaminasyon ve sterilizasyon** | | |
| 1. Sağlık kurumunuz, tıbbi cihazların ve diğer öğelerin/ekipmanın dekontaminasyonu ve sterilizasyonu için özel bir dekontaminasyon alanı ve/veya sterilizasyon departmanı (tesis içinde veya dışında mevcut ve lisanslı bir dekontaminasyon yönetim servisi tarafından işletiliyor) sağlıyor mu? | Hayır, mevcut değil | 0 |
|  | Evet, ancak güvenilir şekilde çalışmıyor | 2,5 |
|  | Evet, güvenilir şekilde çalışıyor | 5 |
| 1. Kullanıma hazır, güvenilir bir şekilde steril ve dezenfekte edilmiş ekipmanınız var mı? | Hayır, haftada ortalama <5 gün kullanılabilir | 0 |
|  | Evet, haftada ortalama ≥ 5 gün veya her gün mevcut, ancak yeterli miktarda değil | 2,5 |
|  | Evet, her gün mevcut, yeterli miktarda | 5 |
| 1. Gerektiğinde tek kullanımlık ürünler mevcut mu? (Ör, enjeksiyon güvenlik cihazları, muayene eldivenleri) | Hayır, mevcut değil | 0 |
|  | Evet, fakat sadece bazen mevcut | 2,5 |
|  | Evet, sürekli olarak mevcut | 5 |
| **Ara toplam puan** | **/100** | |
