# Supplementary material for: Evaluation of the implementation of WHO infection prevention and control core components in Turkish health care facilities: results from a WHO infection prevention and control assessment framework (IPCAF)—based survey
Source: Antimicrob Resist Infect Control. 2023 Feb 13;12:11. doi: 10.1186/s13756-023-01208-0 (PMC9923650; doi:10.1186/s13756-023-01208-0)
Supplement: Supplementary file 3 — Additional file 3 The features of ICUs and HAIs in ICUs, 2021. [file 13756_2023_1208_MOESM3_ESM.docx]

**Additional File 3**.  **The features of ICUs and HAIs in ICUs, 2021.**

| **Table 1**. Features of ICUs ve HAIs in adult ICUs, 2021 |  | | | | | |
| --- | --- | --- | --- | --- | --- | --- |
| **Features of ICUs** | **Adult ICU** | | | | | |
|  | **TRH** | **UH** | **CH** | **SH** | **PH** | **Total** |
| Number of centers performing intensive care unit surveillance | 27 (42,2%) | 20 (31,3%) | 7 (10,9%) | 7 (10,9%) | 3 (4,7%) | 64 (100%) |
| Number of hospital beds | 17824 | 16052 | 7033 | 3263 | 673 | 44845 |
| Number of hospitalized patients | 851370 | 814715 | 323527 | 130409 | 73198 | 2193219 |
| Number of ICU beds | 2302 | 1614 | 1265 | 289 | 109 | 5579 |
| Number of nurses per day bed, Median (IQR) | 0,50 (0,0) | 0,50 (0,17) | 0,50 (0,0) | 0,50 (0,14) | 0,50 (-) | 0,50 (0,05) |
| Number of nurses per bed per night, (Median (IQR) | 0,50 (0,06) | 0,34 (0,17) | 0,50 (0,17) | 0,50 (0,17) | 1,0 (-) | 0,50 (0,17) |
| Number of patients hospitalized in the ICU | 115807 | 68325 | 47213 | 12200 | 3494 | 247039 |
| Patient days in ICU | 547231 | 331327 | 269546 | 68199 | 24708 | 1241011 |
| Number of infected patients in ICU | 12399 | 2679 | 1970 | 366 | 962 | 18276 |
| Number of nosocomial infections in ICU | 4885 | 3521 | 2510 | 461 | 592 | 11969 |
| Number, Non-device-associated nosocomial infection in ICU | 1155 | 976 | 363 | 165 | 502 | 3161 |
| Number, Device-associated nosocomial infection in ICU | 3730 | 2545 | 2147 | 296 | 90 | 8808 |
| **Device utilization ratio** | | | | | | |
| Central line utilization ratio | 0,45 | 0,47 | 0,47 | 0,38 | 0,48 | 0,46 |
| Urinary catheter utilization ratio | 0,87 | 0,89 | 0,86 | 0,91 | 0,91 | 0,88 |
| Ventilator utilization ratio | 0,37 | 0,36 | 0,31 | 0,35 | 0,5 | 0,36 |
| **Incidence density of device-associated HAI (per 1000 device days)** | | | | | | |
| CLABSI | 7,5 | 7,2 | 7,6 | 3,3 | 0,8 | 7,1 |
| CA-UTI | 0,84 | 1,6 | 1,9 | 0,97 | 1,7 | 0,13 |
| VAP | 2,8 | 6,9 | 6,7 | 6 | 3,2 | 4,8 |
| VAE | 4,6 | 0,86 | 2,3 | 0,42 | 0,24 | 2,8 |
| **Non-device-associated HAI in ICU (%)** | | | | | | |
| BSI | 58,1 | 41 | 68,3 | 37,6 | 22,3 | 47 |
| UTI | 8,8 | 5,8 | 16,3 | 17,6 | 18,5 | 10,7 |
| Pneumonia | 22,7 | 34,3 | 3,6 | 33,9 | 50,4 | 29,1 |
| LRI | 1 | 0,5 | 0,8 | 7,9 | 3,8 | 1,6 |
| Skin and soft tissue infection | 5,2 | 8,6 | 8,8 | 3 | 0,8 | 5,9 |
| Bone and joint infection | 0,4 | 0 | 0 | 0 | 1,2 | 0,3 |
| Cardiovascular system infection | 0,2 | 0,4 | 0 | 0 | 0 | 0,2 |
| Eye, ear, nose, throat and mouth infections | 0,2 | 1,7 | 0 | 0 | 0 | 0,6 |
| CNI | 2,3 | 3 | 1,1 | 0 | 0,8 | 2 |
| GI | 0,5 | 2,1 | 1,1 | 0 | 2 | 1,3 |
| RI | 0,1 | 0,4 | 0 | 0 | 0,2 | 0,2 |
| Systemic infections | 0 | 0,1 | 0 | 0 | 0 | 0,03 |
| **Non-device-associated nosocomial infection in ICU (per 1000 patients days)** | | | | | | |
| BSI | 1,2 | 1,3 | 0,9 | 0,9 | 4,5 | 0,12 |
| UTI | 0,2 | 0,18 | 0,2 | 0,4 | 3,8 | 0,3 |
| Pneumonia | 0,5 | 1,1 | 0,04 | 0,8 | 10,2 | 0,8 |
| LRI | 0,02 | 0,02 | 0,01 | 0,2 | 0,8 | 0,04 |
| Skin and soft tissue infection | 0,1 | 0,3 | 0,1 | 0,07 | 0,16 | 0,15 |
| Bone and joint infection | 0,009 | 0 | 0 | 0 | 0,2 | 0,009 |
| Cardiovascular system infection | 0,004 | 0,01 | 0 | 0 | 0 | 0,005 |
| Eye, ear, nose, throat and mouth infections | 0,004 | 0,05 | 0 | 0 | 0 | 0,015 |
| CNI | 0,05 | 0,09 | 0,02 | 0 | 0,2 | 0,05 |
| GI | 0,01 | 0,06 | 0,02 | 0 | 0,4 | 0,03 |
| RI | 0,002 | 0,01 | 0 | 0 | 0,04 | 0,005 |
| Systemic infections | 0 | 0,003 | 0 | 0 | 0 | 0,0008 |
| *ICUs* Intensive care units,  *HAI*s Health care-associated infections, *TRH* training and research hospital, *UH* university hospital, *CH* city hospital, *SH* state hospital, *PH* private hospital, *CLABSI* Central line- associated blood stream infections, *CA-UTI* Catheter-associated urinary tract infection, *VAE* Ventilator-associated event, *VAP* Ventilator-associated pneumonia, *BSI* blood stream infections*, UTI* urinary tract infection*, LRI* Lower respiratory tract infection, other than pneumonia, *CNI* Central nervous system infection, *GI* Gastrointestinal system infection, *RI* Reproductive tract infection | | | | | | |

| **Table 2.** Features of ICUs ve HAIs in neonatal ICUs, 2021 |  | | | | | |
| --- | --- | --- | --- | --- | --- | --- |
|  | **Neonatal ICU** | | | | | |
| **Features of ICUs** | **TRH** | **UH** | **CH** | **SH** | **PH** | **Total** |
| Number of centers performing aintensive care unit surveillance | 14 (29,8%) | 21 (44,7%) | 6 (12,8%) | 3 (6,4%) | 3 (6,4%) | 47 (100%) |
| Number of hospital beds | 12323 | 16278 | 6579 | 2063 | 673 | 37916 |
| Number of hospitalized patients | 583282 | 825259 | 319437 | 85568 | 73198 | 1886744 |
| Number of ICU beds | 575 | 647 | 190 | 134 | 51 | 1597 |
| Number of nurses per day bed, Median (IQR) | 0,46 (0,17) | 0,28 (0,25) | 0,33 (0,19) | 0,55 (-) | 0,60 (-) | 0,33 (0,25) |
| Number of nurses per bed per night, (Median (IQR) | 0,38 (0,17) | 0,30 (0,12) | 0,33 (0,16) | 0,55 (-) | 0,60 (-) | 0,33 (0,25) |
| Number of patients hospitalized in the ICU | 17169 | 14298 | 7944 | 10154 | 1438 | 51003 |
| Patient days in ICU | 166995 | 142027 | 48481 | 35353 | 9355 | 402211 |
| Number of infected patients in ICU | 311 | 425 | 144 | 27 | 72 | 979 |
| Number of nosocomial infections in ICU | 400 | 488 | 238 | 27 | 84 | 1237 |
| Number, Non-device-associated nosocomial infection in ICU | 195 | 308 | 50 | 17 | 79 | 649 |
| Number, Device-associated nosocomial infection in ICU | 205 | 180 | 188 | 10 | 5 | 588 |
| **Device utilization ratio** | | | | | | |
| Central line utilization ratio | 0,15 | 0,17 | 0,19 | 0,13 | 0,32 | 0,2 |
| Urinary catheter utilization ratio | 0,006 | 0,008 | 0,007 | 0,001 | 0,027 | 0,007 |
| Ventilator utilization ratio | 0,17 | 0,18 | 0,12 | 0,19 | 0,41 | 0,17 |
| **Incidence density of device-associated HAI (per 1000 device days)** | | | | | | |
| CLABSI | 7,03 | 4,4 | 16,3 | 2,2 | 1,3 | 6,9 |
| CA-UTI | 1,94 | 2,6 | 8,02 | 0 | 0 | 2,8 |
| VAP | 0,96 | 2,9 | 5,9 | 0 | 0,3 | 1,9 |
| VAE |  |  |  |  |  |  |
| **Non-device-associated HAI in ICU (%)** | | | | | | |
| BSI | 69,2 | 43,8 | 28 | 100 | 82,3 | 56,4 |
| UTI | 4,6 | 13,3 | 8 | 0 | 17,7 | 10,5 |
| Pneumonia | 1 | 6,8 | 4 | 0 | 0 | 3,9 |
| LRI | 0 | 0 | 2 | 0 | 0 | 0,2 |
| Skin and soft tissue infection | 3,1 | 3,6 | 6 | 0 | 0 | 3,1 |
| Bone and joint infection | 0 | 0 | 0 | 0 | 0 | 0 |
| Cardiovascular system infection | 0,5 | 0 | 0 | 0 | 0 | 0,2 |
| Eye, ear, nose, throat and mouth infections | 2,1 | 10,1 | 6 | 0 | 0 | 5,9 |
| CNI | 11,8 | 7,1 | 18 | 0 | 0 | 8,3 |
| GI | 5,6 | 12,7 | 28 | 0 | 0 | 9,9 |
| RI | 0 | 0 | 0 | 0 | 0 | 0 |
| Systemic infections | 0,5 | 1 | 0 | 0 | 0 | 0,6 |
| **Non-device-associated nosocomial infection in ICU (per 1000 patients days)** | | | | | | |
| BSI | 0,8 | 0,95 | 0,3 | 0,5 | 6,9 | 0,9 |
| UTI | 0,05 | 0,3 | 0,08 | 0 | 1,5 | 0,2 |
| Pneumonia | 0,01 | 0,2 | 0,04 | 0 | 0 | 0,06 |
| LRI | 0 | 0 | 0,02 | 0 | 0 | 0,003 |
| Skin and soft tissue infection | 0,03 | 0,08 | 0,06 | 0 | 0 | 0,05 |
| Bone and joint infection | 0 | 0 | 0 | 0 | 0 | 0 |
| Cardiovascular system infection | 0,006 | 0 | 0 | 0 | 0 | 0,003 |
| Eye, ear, nose, throat and mouth infections | 0,02 | 0,2 | 0,06 | 0 | 0 | 0,1 |
| CNI | 0,1 | 0,2 | 0,2 | 0 | 0 | 0,1 |
| GI | 0,07 | 0,3 | 0,3 | 0 | 0 | 0,2 |
| RI | 0 | 0 | 0 | 0 | 0 | 0 |
| Systemic infections | 0,006 | 0,02 | 0 | 0 | 0 | 0,01 |
| *ICUs* Intensive care units,  *HAI*s Health care-associated infections, *TRH* training and research hospital, *UH* university hospital, *CH* city hospital, *SH* state hospital, *PH* private hospital, *CLABSI* Central line- associated blood stream infections, *CA-UTI* Catheter-associated urinary tract infection, *VAE* Ventilator-associated event, *VAP* Ventilator-associated pneumonia, *BSI* blood stream infections*, UTI* urinary tract infection*, LRI* Lower respiratory tract infection, other than pneumonia, *CNI* Central nervous system infection, *GI* Gastrointestinal system infection, *RI* Reproductive tract infection | | | | | | |

| **Table 3.** Features of ICUs ve HAIs in pediatric ICU, 2021 |  | | | | | |
| --- | --- | --- | --- | --- | --- | --- |
|  | **Pediatric ICU** | | | | | |
| **Features of ICUs** | **TRH** | **UH** | **CH** | **SH** | **PH** | **Total** |
| Number of centers performing aintensive care unit surveillance | 10 (26,3%) | 20 (52,6%) | 6 (15,8%) | 1 (2,6%) | 1 (2,6%) | 38 (100%) |
| Number of hospital beds | 9448 | 16128 | 6579 | 665 | 242 | 33062 |
| Number of hospitalized patients | 406111 | 823385 | 319437 | 43823 | 42347 | 1635103 |
| Number of ICU beds | 137 | 260 | 151 | 26 | 6 | 580 |
| Number of nurses per day bed, Median (IQR) | 0,50 (0,0) | 0,46 (0,17) | 0,50 (0,0) | X | X | 0,50 (0,15) |
| Number of nurses per bed per night, (Median (IQR) | 0,50 (0,13) | 0,33 (0,17) | 0,50 (0,04) | X | X | 0,50 (0,17) |
| Number of patients hospitalized in the ICU | 3848 | 6127 | 5893 | 221 | 185 | 16274 |
| Patient days in ICU | 31474 | 53594 | 46485 | 9441 | 1805 | 142799 |
| Number of infected patients in ICU | 121 | 256 | 221 | 48 | 3 | 649 |
| Number of nosocomial infections in ICU | 134 | 383 | 238 | 48 | 3 | 806 |
| Number, Non-device-associated nosocomial infection in ICU | 14 | 129 | 116 | 0 | 0 | 259 |
| Number, Device-associated nosocomial infection in ICU | 120 | 254 | 122 | 48 | 3 | 547 |
| **Device utilization ratio** | | | | | | |
| Central line utilization ratio | 0,63 | 0,5 | 0,4 | 0,7 | 0,6 | 0,5 |
| Urinary catheter utilization ratio | 0,4 | 0,3 | 0,3 | 0,2 | 0,2 | 0,3 |
| Ventilator utilization ratio | 0,53 | 0,5 | 0,4 | 0,95 | 0,6 | 0,5 |
| **Incidence density of device-associated nosocomial infection (per 1000 device days)** | | | | | | |
| CLABSI | 3,7 | 6,7 | 4,9 | 2,4 | 0 | 4,9 |
| CA-UTI | 0,3 | 1,3 | 1,03 | 0 | 0 | 0,9 |
| VAP | 2,5 | 2 | 1,05 | 3,7 | 3 | 2,1 |
| VAE |  |  |  |  |  |  |
| **Non-device-associated HAI in ICU (%)** | | | | | | |
| BSI | 28,6 | 35,7 | 56 | 0 | 0 | 44,4 |
| UTI | 0 | 14,7 | 12,1 | 0 | 0 | 12,7 |
| Pneumonia | 21,4 | 15,5 | 7,8 | 0 | 0 | 12,4 |
| LRI | 0 | 0,8 | 3,5 | 0 | 0 | 1,9 |
| Skin and soft tissue infection | 0 | 10,1 | 12,1 | 0 | 0 | 10,4 |
| Bone and joint infection | 0 | 0 | 0 | 0 | 0 | 0 |
| Cardiovascular system infection | 7,1 | 0,8 | 0 | 0 | 0 | 0,8 |
| Eye, ear, nose, throat and mouth infections | 0 | 5,4 | 1,7 | 0 | 0 | 3,5 |
| CNI | 14,3 | 7 | 5,2 | 0 | 0 | 6,6 |
| GI | 0 | 2,3 | 1,7 | 0 | 0 | 1,9 |
| RI | 0 | 0 | 0 | 0 | 0 | 0 |
| Systemic infections | 0 | 3,1 | 0 | 0 | 0 | 1,5 |
| **Non-device-associated HAI in ICU (per 1000 patients days)** | | | | | | |
| BSI | 0,13 | 0,9 | 1,4 | 0 | 0 | 0,8 |
| UTI |  | 0,4 | 0,3 | 0 | 0 | 0,2 |
| Pneumonia | 0,1 | 0,4 | 0,2 | 0 | 0 | 0,2 |
| LRI |  | 0,02 | 0,1 | 0 | 0 | 0,04 |
| Skin and soft tissue infection |  | 0,2 | 0,3 | 0 | 0 | 0,2 |
| Bone and joint infection |  |  |  | 0 | 0 | 0 |
| Cardiovascular system infection | 0,03 | 0,02 |  | 0 | 0 | 0,01 |
| Eye, ear, nose, throat and mouth infections |  | 0,1 | 0,04 | 0 | 0 | 0,06 |
| CNI | 0,06 | 0,2 | 0,1 | 0 | 0 | 0,1 |
| GI |  | 0,06 | 0,04 | 0 | 0 | 0,04 |
| RI |  |  |  | 0 | 0 | 0 |
| Systemic infections |  | 0,08 |  | 0 | 0 | 0,03 |
| *ICUs* Intensive care units,  *HAI*s Health care-associated infections, *TRH* training and research hospital, *UH* university hospital, *CH* city hospital, *SH* state hospital, *PH* private hospital, *CLABSI* Central line- associated blood stream infections, *CA-UTI* Catheter-associated urinary tract infection, *VAE* Ventilator-associated event, *VAP* Ventilator-associated pneumonia, *BSI* blood stream infections*, UTI* urinary tract infection*, LRI* Lower respiratory tract infection, other than pneumonia, *CNI* Central nervous system infection, *GI* Gastrointestinal system infection, *RI* Reproductive tract infection | | | | | | |
