# Supplementary material for: Evaluation of the implementation of WHO infection prevention and control core components in Turkish health care facilities: results from a WHO infection prevention and control assessment framework (IPCAF)—based survey
Source: Antimicrob Resist Infect Control. 2023 Feb 13;12:11. doi: 10.1186/s13756-023-01208-0 (PMC9923650; doi:10.1186/s13756-023-01208-0)
Supplement: Supplementary file 4 — Additional file 4 Certain IPC components are linked to health care-associated infections. [file 13756_2023_1208_MOESM4_ESM.docx]

**Additional File 4.** Certain IPC components are linked to health care-associated infections.

| **Table 1.** Certain IPC components are linked to VAP in intensive care units | | | | | | | | |
| --- | --- | --- | --- | --- | --- | --- | --- | --- |
|  | **VAE** | | **VAP** | | | | | |
|  | **Adult ICU** | | **Neonatal ICU** | | **Pediatric ICU** | | **Adult ICU** | |
|  | Median (IQR) | *p* | Median (IQR) | *p* | Median (IQR) | *p* | Median (IQR) | *p* |
| **Hospital** | | | | | | | | |
| Training and Research Hospitals | 1.68 (6.18) | 0.1 | 0.60 (2.1) | 0.26 | 1.91 (4.3) | 0.49 | 0.27 (3.6) | 0.27 |
| University Hospitals | 0.00 (4.47) |  | 0.00 (4.92) |  | 1.19 (2.85) |  | 5.61 (10.37) |  |
| City Hospitals | 0.60 (15.3) |  | 6.37 (13.2) |  | 0.76 (14.5) |  | 0.80 (17.3) |  |
| State Hospitals | 0.00 (1.07) |  | 0.00 (0.00) |  | - |  | 8.63 (24.3) |  |
| Private Hospitals | 0.00 (-) |  | 0.00 (-) |  | - |  | 0.56 (-) |  |
| **Frequency of Infection Control Committe meeting** | | | | | | | | |
| Once a month | - | 0.86 | 0.00 (0.00) | 0.29 | 0.00 (0.00) | 0.12 | - | 0.78 |
| Once in three months | 0.00 (4.94) |  | 0.00 (2.86) |  | 1.61 (3.33) |  | 2.39 (9.16) |  |
| Less than once in three months | 0.38 (4.92) |  | 4.17 (-) |  | 2.68 (-) |  | 1.16 (8.78) |  |
| **Cerficated infection control doctor** | | | | | | | | |
| Yes | 0.47 (5.54) | 0.15 | 0.00 (3.25) | 0.97 | 1.61 (3.30) | 0.13 | 1.01 (7.61) | **0.02** |
| No | 0.00 (0.00) |  | 0.00 (-) |  | 0.00 (0.00) |  | 12.91 (-) |  |
| **Cerficated infection control nurse** | | | | | | | | |
| Yes | 0.00 (3.81) | 0.34 | 0.00 (4.35) | 0.28 | 1.24 (3.30) | 0.82 | 1.34 (7.79) | 0.27 |
| No | 4.94 (20.8) |  | 0.00 (0.22) |  | 1.48 (-) |  | 9.07 (29.1) |  |
| **Direct observation of hand hygiene frequency** | | | | | | | | |
| Once a day | 0.74 (7.01) | 0.60 | 0.30 (5.35) | 0.52 | 2.22 (3.93) | 0.36 | 2.95 (9.91) | 0.81 |
| Three times a week | 0.19 (3.33) |  | 2.76 (8.80) |  | 0.74 (-) |  | 1.34 (12.9) |  |
| Twice a week | 0.34 (3.80) |  | 0.00 (5.19) |  | 0.78 (3.18) |  | 0.79 (6.89) |  |
| Once a week | 0.00 (1.36) |  | 0.12 (2.11) |  | 2.35 (2.79) |  | 1.35 (4.61) |  |
| Less than once a week | 0.00 (7.45) |  | 0.00 (0.44) |  | 0.00 (-) |  | 3.81 (12.1) |  |
| **Feedback for hand hygiene compliance frequency** | | | | | | | | |
| Once a month | 0.38 (2.54) | 0.39 | 0.00 (0.00) | 0.96 | 0.00 (3.2) | **0.02** | 2.91 (28.1) | 0.16 |
| Once in three months | 0.33 (6.17) |  | 0.21 (4.42) |  | 1.24 (2.91) |  | 1.18 (8.74) |  |
| Less than once in three months | 0.00 (0.00) |  | 1.95 (-) |  | 4.45 (-) |  | 9.64 (-) |  |
| None | 0.30 (-) |  | - |  | - |  | 1.48 (-) |  |
| **Frequency of report of antiseptic using** | | | | | | | | |
| Once a month | 0.00 (-) | 0.63 | 0.00 (0.00) | 0.20 | 0.00 (0.00) | 0.06 | 0.54 (-) | 0.17 |
| Once in three months | 0.47 (5.76) |  | 0.12 (4.41) |  | 2.21 (3.30) |  | 1.01 (9.1) |  |
| None | 0.00 (5.28) |  | 0.00 (12.7) |  | 0.48 (1.98) |  | 6.75 (5.98) |  |
| **Structure of intensive care unit** | | | | | | | | |
| Fully isolated room | 4.94 (10.6) | **0.02** | 1.27 (9.34) | 0.18 | 0.95 (5.53) | 0.54 | 1.01 (9.27) | 0.41 |
| Mixed or fully open | 0.00 (2.14) |  | 0.00 (2.34) |  | 1.15 (2.85) |  | 2.08 (9.07) |  |
| **Compliance with the target of at least one infection control nurse in 250 beds** | | | | | | | | |
| Yes | 2.24 (7.01) | 0.14 | 0.00 (0.09) | 0.10 | 1.42 (2.94) | 0.91 | 0.58 (9.07) | 0.52 |
| No | 0.00 (3.03) |  | 0.28 (4.45) |  | 1.15 (3.30) |  | 2.55 (9.21) |  |
| **Nurse per bed in the daytime** | | | | | | | | |
| < 0.5 | 0.00 (0.46) | **0.03** | 0.00 (2.10) | 0.35 | 3.50 (7.00) | 0.70 | 6.75 (11.7) | 0.08 |
| **≥ 0.5** | 0.71 (5.85) |  | 0.58 (4.46) |  | 1.50 (5.00) |  | 0.71 (7.6) |  |
| **Nurse per bed at night** | | | | | | | | |
| < 0.5 | 0.00 (5.85) | 0.57 | 0.00 (5.4) | 0.58 | 2.00 (7.00) | 1.00 | 5.56 (12.5) | 0.29 |
| ≥ 0.5 | 0.60 (4.66) |  | 0.00 (2.1) |  | 1.50 (6.00) |  | 1.01 (7.43) |  |
| **Number of hospital bed** | | | | | | | | |
| ≤ 500 | 0.30 (5.23) | 0.84 | 0.00 (4.46) | 0.12 | 0.00 (2.00) | 0.33 | 0.56 (8.63) | 0.20 |
| 501 - 1000 | 0.50 (4.49) |  | 0.12 (4.46) |  | 1.50 (5.00) |  | 0.74 (5.78) |  |
| >1001 | 0.00 (5.26) |  | 3.39 (6.63) |  | 4.00 (8.00) |  | 6.06 (11.1) |  |
| **Sink-to-bed ratio** | | | | | | | | |
| 1.00 | 3.22 (21.98) | 0.23 | 1.95 (-) | 0.67 | 1.19 (5.32) | 0.62 | 5.46 (35.12) | 0.42 |
| <1.00 | 0.00 (3.79) |  | 0.00 (3.71) |  | 1.05 (2.90) |  | 1.71 (8.00) |  |
| *VAE* Ventilator-associated event, *VAP* Ventilator-associated pneumonia, *IPC* Infection prevention and control, *IQR* Interquantile range | | | | | | | | |

| **Table 2.** Certain IPC components are linked to CLABSI in intensive care units | | | | | | |
| --- | --- | --- | --- | --- | --- | --- |
|  | **CLABSI** | | | | | |
|  | **Neonatal ICU** | | **Pediatric ICU** | | **Adult ICU** | |
|  | Median (IQR) | *p* | Median (IQR) | *p* | Median (IQR) | *p* |
| **Hospital** | | | | | | |
| Training and Research Hospitals | 4.83 (7.7) | 0.53 | 1.27 (7.1) | 0.36 | 4.80 (9.2) | **0.03** |
| University Hospitals | 2.37 (6.95) |  | 2.02 (7.66) |  | 5.80 (9.06) |  |
| City Hospitals | 6.69 (32.6) |  | 8.20 (10.4) |  | 4.58 (3.4) |  |
| State Hospitals | 1.22 (-) |  | - |  | 1.86 (6.77) |  |
| Private Hospitals | 0.68 (-) |  | - |  | 0.00 (-) |  |
| **Frequency of Infection Control Committe meeting** | | | | | | |
| Once a month | 0.88 (-) | 0.31 | 0.59 (-) | 0.22 | - | 0.28 |
| Once in three months | 2.66 (8.50) |  | 5.03 (9.51) |  | 4.81 (7.63) |  |
| Less than once in  three months | 1.10 (-) |  | 3.23 (-) |  | 4.22 (9.39) |  |
| **Cerficated infection control doctor** | | | | | | |
| Yes | 1.98 (7.23) | 0.15 | 3.23 (7.77) | 0.43 | 4.81 (7.49) | 0.72 |
| No | 5.06 (-) |  | 0.96 (-) |  | 4.39 (-) |  |
| **Cerficated infection control nurse** | | | | | | |
| Yes | 2.52 (7.70) | 0.21 | 3.23 (7.55) | 0.13 | 4.78 (5.73) | 0.33 |
| No | 0.88 (-) |  | 0.29 (-) |  | 12.2 (92.5) |  |
| **Direct observation of hand hygiene frequency** | | | | | | |
| Once a day | 1.36 (7.14) | 0.22 | 1.55 (7.77) | 0.57 | 4.97 (8.51) | 0.87 |
| Three times a week | 1.66 (6.32) |  | 6.09 (-) |  | 6.01 (10.2) |  |
| Twice a week | 0.00 (9.52) |  | 3.23 (6.27) |  | 4.22 (6.18) |  |
| Once a week | 6.99 (10.7) |  | 2.35 (12.3) |  | 4.30 (3.33) |  |
| Less than once a week | 5.13 (1.05) |  | 7.56 (-) |  | 4.76 (9.49) |  |
| **Feedback for hand hygiene compliance frequency** | | | | | | |
| Once a month | 0.44 (2.00) | 0.06 | 0.59 (3.81) | 0.06 | 1.28 (45.9) | 0.17 |
| Once in three months | 2.43 (7.77) |  | 2.35 (8.31) |  | 5.27 (7.48) |  |
| Less than once in three months | 7.43 (-) |  | 8.35 (-) |  | 6.34 (-) |  |
| None | - |  | - |  | 2.79 (-) |  |
| **Frequency of report of antiseptic using** | | | | | | |
| Once a month | 0.88 (-) | 0.56 | 0.59 (-) | 0.13 | 0.00 (-) | **0.02** |
| Once in three  months | 1.70 (9.00) |  | 2.35 (7.29) |  | 4.81 (7.66) |  |
| None | 4.37 (6.45) |  | 7.47 (7.77) |  | 5.04 (5.39) |  |
| **Structure of intensive care unit** | | | | | | |
| Fully isolated room | 6.98 (12.5) | 0.49 | 6.09 (6.52) | 0.27 | 6.01 (9.10) | 0.13 |
| Mixed or fully open | 1.98 (7.02) |  | 1.73 (12.8) |  | 4.39 (6.01) |  |
| **Compliance with the target of at least one infection control nurse in 250 beds** | | | | | | |
| Yes | 0.00 (2.37) | 0.12 | 5.50 (11.5) | 0.63 | 4.64 (11.7) | 0.73 |
| No | 2.63 (7.13) |  | 1.73 (7.56) |  | 4.89 (5.73) |  |
| **Nurse per bed in the daytime** | | | | | | |
| < 0.5 | 2.37 (7.43) | 0.80 | 10.5 (23.0) | 0.12 | 4.68 (7.20) | 0.90 |
| ≥ 0.5 | 1.82 (7.80) |  | 2.00 (13.0) |  | 4.80 (7.72) |  |
| **Nurse per bed at night** | | | | | | |
| < 0.5 | 2.49 (8.04) | 0.39 | 6.00 (18.0) | 0.65 | 4.68 (7.95) | 0.76 |
| ≥ 0.5 | 1.21 (6.38) |  | 4.50 (15.0) |  | 4.80 (7.13) |  |
| **Number of hospital bed** | | | | | | |
| ≤ 500 | 0.00 (2.37) | 0.099 | 0.50 (1.0) | **0.006** | 2.17 (7.10) | **0.02** |
| 501 - 1000 | 3.74 (9.05) |  | 4.50 (1.53) |  | 5.12 (10.62) |  |
| >1001 | 3.54 (6.78) |  | 10.0 (22.0) |  | 5.87 (6.40) |  |
| **Sink-to-bed ratio** | | | | | | |
| 1.00 | 9.52 (-) | 0.47 | 6.77 (7.33) | 0.27 | 6.79 (17.88) | 0.15 |
| <1.00 | 2.18 (7.09) |  | 2.04 (9.32) |  | 4.72 (7.63) |  |
| *CLABSI* Central line- associated blood stream infections, *IPC* Infection prevention and control, *IQR* Interquantile range | | | | | | |

| **Table 3.** Certain IPC components are linked to CA-UTI in intensive care units | | | | | | |
| --- | --- | --- | --- | --- | --- | --- |
|  | **CA-UTI** | | | | | |
|  | **Neonatal ICU** | | **Pediatric ICU** | | **Adult ICU** | |
|  | Median (IQR) | *p* | Median (IQR) | *p* | Median (IQR) | *p* |
| **Hospital** | | | | | | |
| Training and Research Hospitals | 0.00 (1.0) | 0.61 | 0.00 (1.0) | 0.85 | 0.68 (1.0) | 0.19 |
| University Hospitals | 0.00 (0.00) |  | 0.00 (1.47) |  | 1.24 (1.47) |  |
| City Hospitals | 0.00 (45.4) |  | 0.26 (3.49) |  | 0.82 (0.66) |  |
| State Hospitals | 0.00 (0.00) |  | - |  | 1.02 (1.93) |  |
| Private Hospitals | 0.00 (-) |  | - |  | 1.22 (-) |  |
| **Frequency of Infection Control Committe meeting** | | | | | | |
| Once a month | 0.00 (-) | 0.47 | 0.00 (0.00) | 0.26 | - | 0.33 |
| Once in three months | 0.00 (0.00) |  | 0.00 (1.03) |  | 1.01 (1.14) |  |
| Less than once in  three months | - |  | 0.73 (-) |  | 0.43 (3.59) |  |
| **Cerficated infection control doctor** | | | | | | |
| Yes | 0.00 (0.00) | **0.01** | 0.00 (1.03) | 0.97 | 0.90 (1.06) | 0.65 |
| No | 45.04 (-) |  | 0.26 (-) |  | 2.15 (-) |  |
| **Cerficated infection control nurse** | | | | | | |
| Yes | 0.00 (1.02) | 0.55 | 0.00 (1.03) | 0.47 | 0.85 (1.06) | **0.001** |
| No | 0.00 (-) |  | 0.00 (0.00) |  | 3.25 (8.5) |  |
| **Direct observation of hand hygiene frequency** | | | | | | |
| Once a day | 0.00 (3.05) | 0.95 | 0.00 (1.62) | 0.82 | 0.85 (1.05) | 0.37 |
| Three times a week | - |  | 0.00 (0.00) |  | 0.58 (1.32) |  |
| Twice a week | 0.00 (0.00) |  | 0.00 (0.63) |  | 1.28 (1.95) |  |
| Once a week | 0.00 (6.94) |  | 1.92 (1.03) |  | 0.79 (1.04) |  |
| Less than once a week | 0.00 (-) |  | 0.00 (-) |  | 0.94 (1.29) |  |
| Feedback for hand hygiene compliance frequency | | | | | | |
| Once a month | 0.00 (0.00) | 0.12 | 0.00 (0.37) | **0.005** | 1.14 (4.18) | 0.35 |
| Once in three months | 0.00 (0.00) |  | 0.00 (0.52) |  | 0.89 (1.26) |  |
| Less than once in three months | - |  | 4.04 (-) |  | 1.15 (-) |  |
| None | - |  | - |  | 0.42 (-) |  |
| **Frequency of report of antiseptic using** | | | | | | |
| Once a month | 0.00 (0.00) | 0.67 | 0.00 (0.00) | 0.23 | 0.23 (-) | 0.34 |
| Once in three months | 0.00 (0.00) |  | 0.00 (0.53) |  | 0.90 (1.62) |  |
| None | 0.00 (9.87) |  | 0.79 (3.02) |  | 1.06 (0.89) |  |
| Structure of intensive care unit | | | | | | |
| Fully isolated room | 0.00 (0.00) | 1.00 | 0.00 (1.30) | 0.97 | 0.90 (1.48) | 0.48 |
| Mixed or fully open | 0.00 (0.00) |  | 0.00 (1.03) |  | 1.01 (1.22) |  |
| Compliance with the target of at least one infection control nurse in 250 beds | | | | | | |
| Yes | 0.00 (0.00) | 0.50 | 0.00 (1.23) | 0.68 | 0.81 (2.24) | 0.65 |
| No | 0.00 (2.03) |  | 0.00 (0.73) |  | 1.01 (1.12) |  |
| Nurse per bed in the daytime | | | | | | |
| < 0.5 | 0.00 (0.00) | 0.39 | 1.00 (2.00) | 0.08 | 1.09 (1.28) | 0.18 |
| ≥ 0.5 | 0.00 (8.98) |  | 0.00 (1.00) |  | 0.83 (1.30) |  |
| **Nurse per bed at night** | | | | | | |
| < 0.5 | 0.00 (0.00) | 0.70 | 0.00 (2.00) | 0.75 | 1.04 (1.42) | 0.40 |
| ≥ 0.5 | 0.00 (4.07) |  | 0.00 (1.00) |  | 0.86 (1.14) |  |
| **Number of hospital bed** | | | | | | |
| ≤ 500 | 0.00 (0.00) | 0.36 | 0.00 (0.0) | 0.046 | 0.60 (1.67) | 0.63 |
| 501 - 1000 | 0.00 (6.34) |  | 0.00 (1.00) |  | 0.96 (1.55) |  |
| >1001 | 0.00 (2.11) |  | 0.50 (2.0) |  | 1.08 (0.77) |  |
| **Sink-to-bed ratio** | | | | | | |
| 1.00 |  | 0.16 | 0.00 (1.11) | 0.92 | 0.98 (6.55) | 0.56 |
| <1.00 | 0.00 (0.00) |  | 0.00 (1.17) |  | 0.87 (1.16) |  |
| *CA-UTI* Catheter-associated urinary tract infection, *IPC* Infection prevention and control, *IQR* Interquantile range | | | | | | |

| **Table 4.** Certain IPC components are linked to Non-Invasive HAI in intensive care units | | | | | | |
| --- | --- | --- | --- | --- | --- | --- |
|  | **Non-Invasive HAI** | | | | | |
|  | **Neonatal ICU** | | **Pediatric ICU** | | **Adult ICU** | |
|  | Median (IQR) | p | Median (IQR) | p | Median (IQR) | p |
| **Hospital** | | | | | | |
| Training and Research Hospitals | 0.60 (1.1) | 0.12 | 0.11 (1.1) | **0.03** | 1.16 (2.3) | 0.25 |
| University Hospitals | 1.53 (4.02) |  | 1.60 (2.4) |  | 1.49 (3.68) |  |
| City Hospitals | 0.97 (1.80) |  | 1.23 (3.67) |  | 0.85 (1.27) |  |
| State Hospitals | 0.00 (0.45) |  | - |  | 2.23 (7.75) |  |
| Private Hospitals | 2.20 (-) |  | - |  | 3.63 (-) |  |
| **Frequency of Infection Control Committe meeting** | | | | | | |
| Once a month | 0.35 (-) | 0.31 | 0.26 (-) | 0.55 | - | 0.58 |
| Once in three months | 0.74 (2.17) |  | 1.14 (2.16) |  | 1.35 (2.89) |  |
| Less than once in  three months | 2.65 (-) |  | 0.68 (-) |  | 1.10 (2.00) |  |
| **Cerficated infection control doctor** | | | | | | |
| Yes | 0.79 (2.25) | 0.64 | 0.80 (1.99) | 0.10 | 1.20 (2.65) | 0.15 |
| No | 2.07 (-) |  | 2.81 (-) |  | 3.35 (-) |  |
| **Cerficated infection control nurse** | | | | | | |
| Yes | 0.80 (2.22) | 1.00 | 1.14 (2.10) | 0.18 | 1.35 (2.70) | 0.33 |
| No | 1.1 (14.96) |  | 0.13 (-) |  | 2.6 (34.5) |  |
| **Direct observation of hand hygiene frequency** | | | | | | |
| Once a day | 1.25 (2.16) | 0.81 | 0.92 (1.41) | 0.94 | 1.68 (2.42) | 0.06 |
| Three times a week | 1.33 (3.00) |  | 2.11 (-) |  | 1.37 (1.55) |  |
| Twice a week | 0.32 (2.74) |  | 0.55 (2.99) |  | 1.92 (11.1) |  |
| Once a week | 0.69 (1.58) |  | 1.57 (2.06) |  | 0.39 (1.73) |  |
| Less than once a week | 0.60 (2.81) |  | 1.19 (-) |  | 0.90 (3.33) |  |
| **Feedback for hand hygiene compliance frequency** | | | | | | |
| Once a month | 0.18 (7.52) | 0.34 | 0.68 (2.10) | 0.23 | 3.66 (19.3) | 0.31 |
| Once in three months | 0.80 (2.10) |  | 1.01 (2.07) |  | 1.27 (2.44) |  |
| Less than once in three months | 1.12 (-) |  | 7.33 (6.80) |  | 5.39 (-) |  |
| None | - |  | - |  | 0.91 (-) |  |
| **Frequency of report of antiseptic using** | | | | | | |
| Once a month | 1.99 (15.2) | 0.23 | 0.26 (-) | 0.45 | 0.45 (-) | 1.00 |
| Once in three months | 0.65 (1.95) |  | 0.88 (2.10) |  | 1.62 (2.73) |  |
| None | 1.95 (4.4) |  | 1.36 (3.77) |  | 1.19 (2.50) |  |
| **Structure of intensive care unit** | | | | | | |
| Fully isolated room | 0.81 (3.75) | 0.95 | 1.09 (3.00) | 0.97 | 0.92 (2.74) | 0.53 |
| Mixed or fully open | 0.83 (1.97) |  | 0.92 (1.95) |  | 1.37 (2.73) |  |
| **Compliance with the target of at least one infection control nurse in 250 beds** | | | | | | |
| Yes | 0.00 (2.35) | 0.20 | 1.30 (1.89) | 0.46 | 1.62 (3.35) | 0.79 |
| No | 0.83 (2.14) |  | 0.65 (1.99) |  | 1.27 (2.22) |  |
| **Nurse per bed in the daytime** | | | | | | |
| < 0.5 | 1.09 (2.36) | 0.96 | 1.28 (1.16) | 0.90 | 1.64 (3.45) | 0.36 |
| ≥ 0.5 | 0.60 (2.32) |  | 0.80 (3.00) |  | 1.28 (2.20) |  |
| **Nurse per bed at night** | | | | | | |
| < 0.5 | 1.25 (2.27) | 0.19 | 1.01 (1.47) | 0.88 | 1.77 (3.53) | 0.12 |
| ≥ 0.5 | 0.56 (1.31) |  | 0.94 (2.99) |  | 1.16 (2.06) |  |
| **Number of hospital bed** | | | | | | |
| ≤ 500 | 0.19 (2.25) | 0.55 | 0.34 (1.73) | 0.47 | 1.06 (3.36) | 0.64 |
| 501 - 1000 | 0.60 (2.23) |  | 1.60 (2.80) |  | 1.25 (2.31) |  |
| >1001 | 1.06 (1.08) |  | 0.77 (1.93) |  | 1.93 (2.40) |  |
| **Sink-to-bed ratio** | | | | | | |
| 1.00 | 1.12 (-) | 0.42 | 1.28 (5.15) | 0.59 | 1.85 (2.72) | 0.88 |
| <1.00 | 0.82 (2.30) |  | 0.80 (2.24) |  | 1.20 (2.89) |  |
| *HAI* Health care-associated infection, *IPC* Infection prevention and control, *IQR* Interquantile range | | | | | | |
